# The use of effective core potentials in Hirshfeld atom refinement: making quantum crystallography faster in *NoSpherA2*

**DOI:** 10.1107/S1600576725000901

**Published:** 2025-03-07

**Authors:** Florian Kleemiss, Florian Meurer, Ilya G. Shenderovich, Michael Bodensteiner

**Affiliations:** a Institute of Inorganic Chemistry RWTH Aachen University Landoltweg 1a 52074Aachen Germany; b Fakultät für Chemie und Pharmazie Universität Regensburg Universitätsstraße 31 93040Regensburg Germany; Warsaw University, Poland

**Keywords:** quantum crystallography, Hirshfeld atom refinement, effective core potentials, *NoSpherA2*, heavy elements

## Abstract

The refinement of structural models against X-ray diffraction data benefits significantly from employing non-spherical scattering factors in terms of precision and accuracy. Tailor-made scattering factors are available from quantum chemical calculations of the electron density using routine calculation packages. In this process, heavy elements in particular pose a difficulty due to their large numbers of electrons which are not involved in chemical bonding. An elegant way to circumvent this is by using effective core potentials. This work presents an approach for the treatment of these missing electrons in advanced structural refinement methods, such as Hirshfeld atom refinement. It also provides examples and benchmarks demonstrating up to a twofold reduction in refinement time without compromising on accuracy.

## Abstract 2

A procedure is presented for the use of effective core potentials during wavefunction-based refinements. The refinement quality of the results is indistinguishable from that of all-electron calculations, with speedups of a factor of 2 for compounds containing heavy elements.

## 1. Introduction

The use of non-spherical scattering factors, for example those obtained by the method of (transferable) multipole models (Hansen & Coppens, 1978 ▸; Jha *et al.*, 2023 ▸; Rybicka *et al.*, 2022 ▸; Dittrich *et al.*, 2013 ▸; Domagała *et al.*, 2012 ▸) or Hirshfeld atom refinement (HAR) (Capelli *et al.*, 2014 ▸; Fugel *et al.*, 2018 ▸; Woińska *et al.*, 2016 ▸; Ruth *et al.*, 2022 ▸), improves the overall quality and certainty of information about a structure obtainable from diffraction data. HAR can accurately and precisely locate hydrogen atoms in light element structures from X-ray data, comparably to neutron diffraction experiments (Woińska *et al.*, 2016 ▸; Novelli *et al.*, 2021 ▸). Previous studies show that refining hydrogen atoms becomes more challenging for heavier elements (Kleemiss *et al.*, 2021 ▸; Woińska *et al.*, 2021 ▸, 2023 ▸). During the calculations required to obtain non-spherical form factors for the refinement, a significant amount of time is spent calculating orbital coefficients using relativistic Hamiltonians for orbitals that show comparatively small deformation, as shown below. The increased time required for wavefunction-based refinement techniques is one of the drawbacks compared with multipole-based techniques, which this work will attempt to address for heavy elements, where the effect is most pronounced.

Within the framework of HAR, the atomic form factor for a scattering vector **k** is obtained from an electron density calculated for a molecule ρ(**r**), for example by *ab initio* calculations, applying the Hirshfeld weighting scheme relying on spherical neutral atomic electron densities (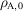) and subsequent Fourier transformation:
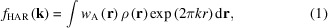
where
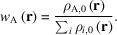
This is in contrast to the independent atom model, where atomic spherically averaged wavefunctions are used to parameterize a set of Gaussian functions as a fit to the form factors.

It has been shown that relativistic effects on the non-spherical scattering factors of atoms are most pronounced in the close vicinity of the core of heavier elements and can potentially affect neighbouring atoms in the structure (Bučinský *et al.*, 2016 ▸; Podhorský *et al.*, 2021 ▸). In addition, effects like electron correlation show similar or even more significant effects close to these heavy atoms, as shown in the work of Bučinský *et al.* (2016 ▸). Therefore, neglecting the impact of relativistic Hamiltonians would introduce considerable errors into the refinement (Bučinský *et al.*, 2016 ▸). However, quantum chemical calculations that consider these effects analytically during the calculation are time consuming. Therefore, different levels of approximations are available within standard software packages. In this work, we will compare the results of refinements using three approaches of scalar relativistic calculations: the zeroth-order relativistic approximation (ZORA) (van Lenthe *et al.*, 1993 ▸, 1994 ▸), the infinite-order relativistic approximation (IORA) (Dyall & van Lenthe, 1999 ▸) and the Douglas–Kroll–Hess second-order Hamiltonian (DKH) (Hess, 1985 ▸; Wolf *et al.*, 2002 ▸, 2004 ▸).

A different approach to tackling this issue in quantum chemical calculations is the application of effective core potentials (ECPs). These were introduced to mimic the effect of the core electrons on the valence shell without explicitly calculating the electrons modelled by the potential (Kahn & Goddard, 1968 ▸; Kahn *et al.*, 1976 ▸; Flores-Moreno *et al.*, 2006 ▸). This allows comparable accuracy of theoretical calculations with much less computational effort.

ECPs are often defined in conjunction with a matching optimized basis set and parameterized by a few key features that characterize the potential:

(i) the number of electrons modelled by the ECP;

(ii) a series of contracted Gaussian functions with ranging angular momenta that model the potential exhibited by the defined number of core electrons that are not explicitly calculated;

(iii) the maximum angular momentum of the ECP expansion.

A problem arises when using this approach to calculate the form factor of atoms within the framework of HAR similar to equation (1): all electrons contribute to the scattering, and the omitted electrons require a treatment that compensates for the missed scattering power in the core regions of heavier elements during the modelling procedure.

Recently, we introduced a model for spherical scattering factors of atoms based on Slater-type orbitals (Kleemiss *et al.*, 2024 ▸). The calculation of contributions to the form factor in this approach is based on the summation of all occupied orbitals, which allows the calculation of individual form factors of subsets of orbitals. Using this approach, it is possible to calculate independently the missing scattering contribution from the core electrons and generate complete form factors.

**Figure 1:**
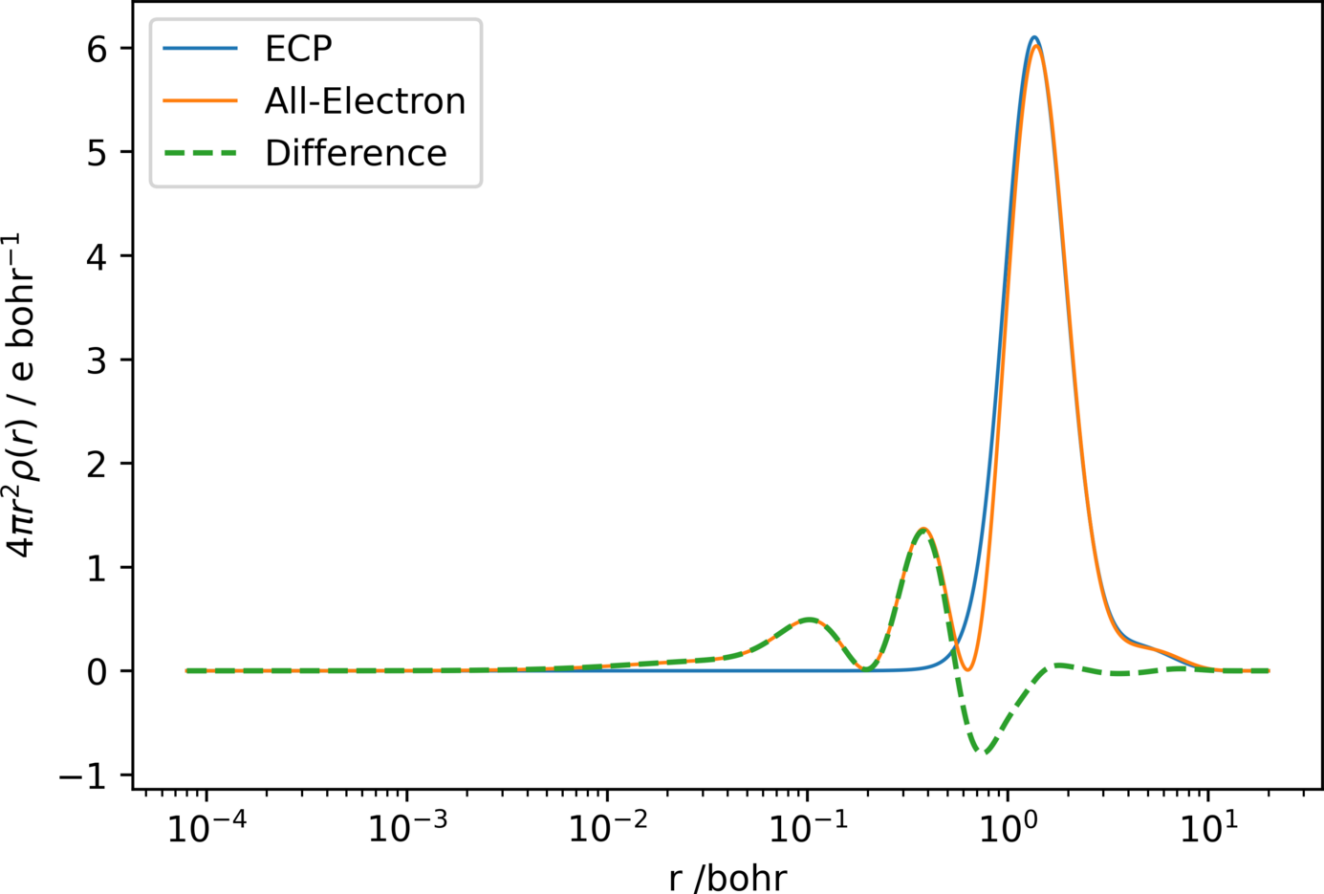
Difference in radial electron distribution function of 3*s*, 3*p*, 3*d*, 4*s*, 4*p* and 5*s* valence orbitals for Rb using def2-TZVPP (Leininger *et al.*, 1996 ▸; Peterson *et al.*, 2003 ▸; Weigend & Ahlrichs, 2005 ▸; Gulde *et al.*, 2012 ▸; Dolg *et al.*, 1989 ▸; Andrae *et al.*, 1990 ▸) (blue), using an all-electron relativistic Jorge-TZVP-DKH calculation (orange) and the difference between the two distributions (green, dashed) against distance from the nucleus on a logarithmic scale.

However, the total electron density obtained would be incorrect if the atomic densities were combined with the valence density obtained by an ECP-based calculation, since the nodal behaviour of the ECP valence orbitals is incorrect (Dolg & Cao, 2024 ▸). The electron density of the remaining orbitals [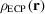] is too low in the near-core region but too high in the mid-distance region, and only becomes reliable at the actual valence distance. This inadequacy in electron density is an intrinsic property of the ECP approach. To visualize this effect, the radial distribution function of the electron density of the valence orbitals of an all-electron calculation [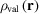] of Rb, the first element in the periodic table with an ECP in the def2 family, and the calculation of the density resulting from an ECP calculation are plotted in Fig. 1 ▸. Only the orbitals of the all-electron calculation that are also included in the ECP basis, *i.e.* removing the 28 lowest energy unrestricted orbitals, were used. This visualization follows the argumentation of Dolg & Cao (2024 ▸).

To account for these differences, we propose a correction function that introduces the density difference of the missing nodal lobes of the valence orbitals to reconstruct correctly the total electron density for calculations of scattering factors.

In this work, we compare electron densities and scattering factors obtained by all-electron calculations using the aforementioned relativistic approximations and ECPs with added calculated core form factors, alongside their application to example heavy element X-ray diffraction structures. A comparison of the calculation times is also included to see if a trade off between time and accuracy might be justified.

## 2. Computation of correction functions and form factors

Depending on the explicit formulation of the ECP, varying valence electron densities and nodal behaviours are obtained. Different correction functions must be used for these different core approaches. Most calculations using ECPs can be divided into small-core and large-core calculations, according to the number of electrons modelled by the ECP. The flexibility of small-core potentials, where the ECP models fewer electrons, compromises the accuracy of the calculation results and the time required to perform them. Therefore, this work will focus on calculating the correction functions and form factors of small-core functions of the def2 family. However, the framework can easily be extended to other core sizes. This family of basis sets has defined ECP parameters from atoms between Rb and Rn (37 ≤ *Z* ≤ 86).

The fit was performed using Gaussian functions centred on the atomic position. The resulting expressions as a function of the fit parameter vectors **a** and **b** and the distance to the atomic position *r* are
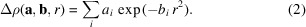


To obtain the parameter vectors **a** and **b**, a target function *t* was defined. To ensure the accuracy of the result in terms of the electron density, the radial electron distribution of increasing moments and the overall number of electrons being conserved, this function was chosen as
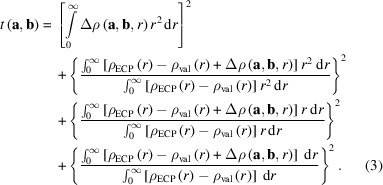


The minimization of this objective function *t* can be considered as a simultaneous least-squares minimization of the electron density difference and its higher moments between 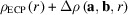 and 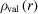 under the restraint that the number of electrons should be conserved. The latter is why the first integral is introduced over the entire radial correction function 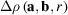. Technically, the integrals were evaluated by numerical integration using fixed-step radial grids with a step size of 0.00008 bohr in a range of 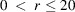
 bohr.

The calculation of spherically averaged electron densities from a non-symmetric wavefunction was implemented in *NoSpherA2* (Kleemiss *et al.*, 2021 ▸), employing averaging on an iteratively growing angular grid for a set of radial points until a predefined convergence criterion of a relative difference between steps of less than 10^−4^ is reached. These calculations were performed on unrestricted ground-state wavefunctions with all electrons obtained using *ORCA* (Version 5.0.4; Neese *et al.*, 2020 ▸) on a level of theory of IORA-MP2/Jorge-TZVP-DKH with finite nucleus approximations and picture change effects taken into account, including spin-orbit coupling (Neese*et al.*, 2020 ▸; Barros *et al.*, 2010 ▸; Camiletti *et al.*, 2008 ▸; Campos & Jorge, 2013 ▸; Campos *et al.*, 2017 ▸; Canal Neto *et al.*, 2005 ▸; De Oliveira *et al.*, 2018 ▸; Machado *et al.*, 2009 ▸; Martins *et al.*, 2016 ▸).

**Figure 2:**
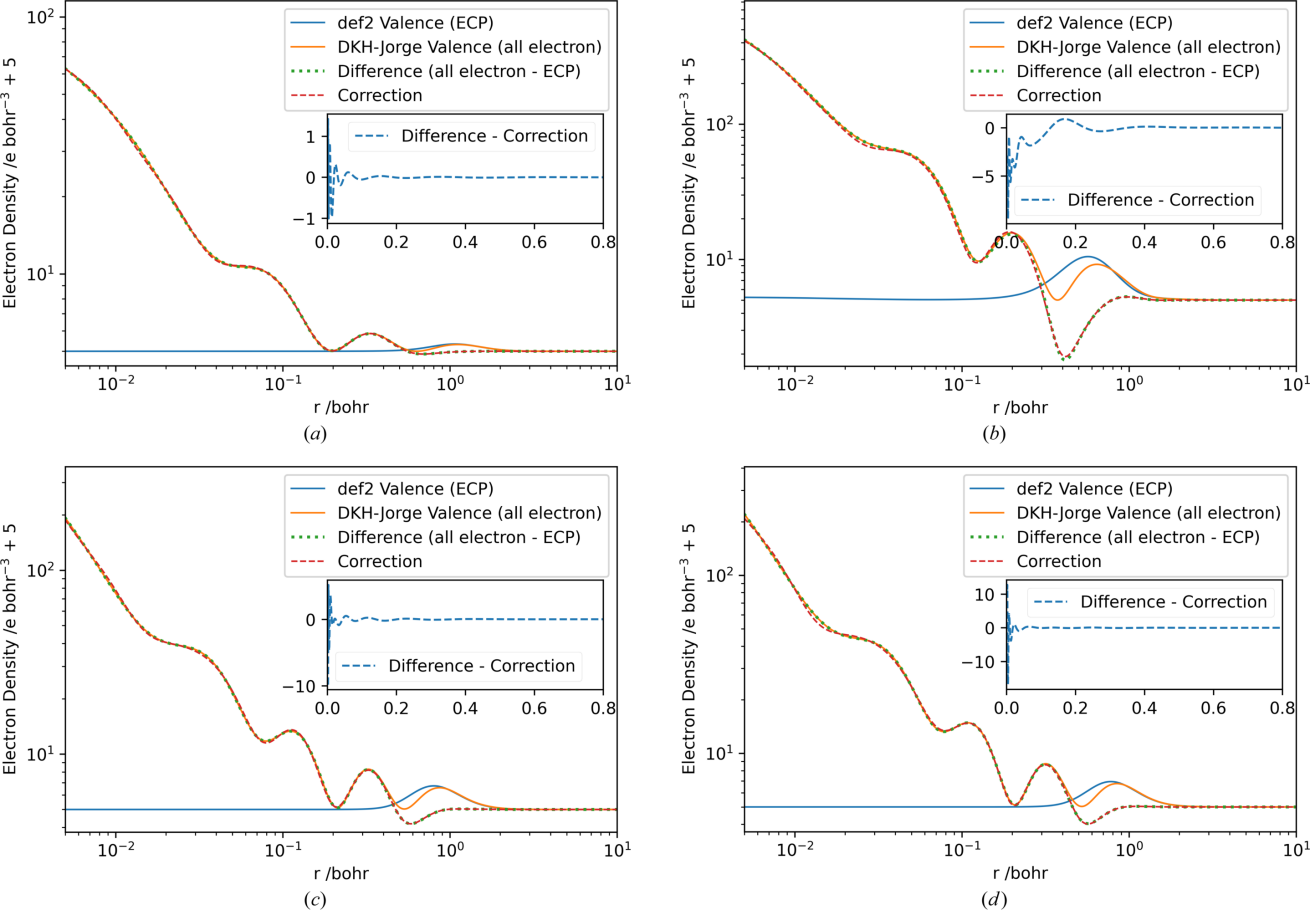
Plots of radial electron density from the def2 wavefunction ρ_ECP_(*r*) (blue), valence electron density from the relativistic Jorge wavefunction ρ_val_(*r*) (orange), the difference between these densities (green, dotted) and the fitted correction function of the difference (red, dashed) for the elements (*a*) Rb, (*b*) I, (*c*) Au and (*d*) Hg on a double logarithmic scale shifted up by an absolute value of 5 to allow logarithmic plotting. Insets contain the absolute difference between the correction function and the density difference to be fitted (blue, dashed).

An initial minimization of the target function *t* [equation (3)] was performed using random initial values for the parameter vectors **a** and **b** employing the ‘minimize’ function in the Python module *SciPy* (Virtanen *et al.*, 2020 ▸). To ensure that the resulting parameters were not just local minima in the parameter space of this fit, a subsequent differential evolution search as implemented in *SciPy* was performed. We consider this approach crucial, because the parameters are highly correlated and a simple minimization would not be able to find the optimal parameters. The parameters for the search were chosen within bounds for 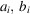 to an interval based on the first values found in the minimum, resulting in a range of 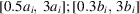, respectively. The sample size for each generation was 15 per parameter, setting the convergence tolerance to 1 × 10^−3^ and the maximum number of generations to 10^6^. This tolerance was chosen because the error function can be understood as a relative error of the three moments of the initial difference between the all-electron and ECP-based calculations. This was judged to be sufficiently accurate, with an error of less than 0.33% on average over all moments of the electron density. If features of the difference function were not correctly included, as judged by plotting fits similar to Figs. 1 and 2, an additional entry was added to the parameters and the fitting routine was repeated.

This procedure was applied to all elements with (37 ≤ *Z* ≤ 86). The resulting function 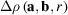 was plotted against the original differences 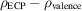 and the electron densities of the valence orbitals (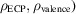 themselves. The final plots are shown in Fig. 2. It was not possible to achieve this convergence limit for all elements within the available computational limits of the local resources. Future updates may improve the errors achieved (see Table S1 in the supporting information). The values obtained are included in the source code of *NoSpherA2* and used when ECP-based calculations are performed.

The resulting functions can compensate for the error in nodal behaviour when evaluating the electron density at a given point to calculate atomic form factors, or can be used to calculate directly the contributions to the form factor. The form factor resulting from the correction function can be calculated according to the procedure described in the sup­porting information of our previous work (Kleemiss *et al.*, 2024 ▸):
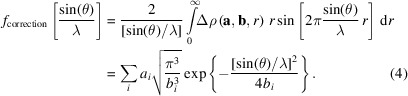


Using equation (4), an overall form factor for an atom of a quantum chemical calculation employing ECPs can be obtained according to a construction of *e.g.* Hirshfeld atoms, supplementing the missing information from the Slater function-based orbitals (Kleemiss *et al.*, 2024 ▸) on the basis of atomic wavefunctions from Koga *et al.* (1999 ▸, 2000 ▸) corresponding to the core that was omitted in the quantum chemical calculation and the correction functions according to equation (3):
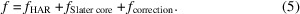


Any source of atomically partitioned valence densities could be adopted with this approach. However, the use of Hirsh­feld atoms has proven to be accurate and robust (Woińska *et al.*, 2023 ▸; Ruth *et al.*, 2022 ▸; Chodkiewicz *et al.*, 2020 ▸).

## 3. Implementation in *NoSpherA2*

Several relativistic approximations are available in quantum chemical software packages such as, for example, *ORCA*, *pySCF* or *Tonto* (Neese *et al.*, 2020 ▸; Jayatilaka & Grimwood, 2003 ▸; Sun *et al.*, 2018 ▸). Within this study, we will focus on implementations available in *ORCA*. Therefore, the selection of a scalar relativistic DKH Hamiltonian has been extended by a possible choice between ZORA, DKH and IORA. The selection is automatically applied during the generation of input files and the calculations are performed accordingly. Instructions for manual control are given in the supporting information.

The choice of using an ECP is included in the selection of a basis set when a heavy element is present in the structure: the def2 family of basis sets (Leininger *et al.*, 1996 ▸; Peterson *et al.*, 2003 ▸; Weigend & Ahlrichs, 2005 ▸; Gulde *et al.*, 2012 ▸; Dolg *et al.*, 1989 ▸; Andrae *et al.*, 1990 ▸) has been included for elements up to Rn. Heavier elements will require a different basis set, since the def2 family is not parameterized for elements heavier than Rn.

Note that a combination of ECPs with a scalar relativistic Hamiltonian is unreasonable, since the relativistic effect is already taken into account by the coefficients of the potentials.

When calculating the scattering factors from a wavefunction given to *NoSpherA2*, the additional command line keyword -ECP will enable two additional steps during the calculation:

(i) inclusion of core electron densities for atoms with ECPs from the Slater densities as implemented previously (Kleemiss *et al.*, 2024 ▸), and

(ii) addition of the correction function for atoms with ECPs, both in the calculation of the statistics of the non-spherical density printed after partitioning and in the calculation of the form factors.

An integer number given as a command line argument can be used to select different modes to switch between, corresponding to predefined sets of ECPs. Mode (i) defines all atoms according to the ECPs present in the def2 family of basis sets. Further modes are planned to extend this to additional basis sets, but this is not implemented at present. An updated table summarizing the available modes is printed using the -help command.

The complete form factors are printed to the resulting .tsc/.tscb file, so no additional steps are required to calculate the structure factors.

## 4. Results and discussion

The resulting fitting functions, the corresponding scattering factors and the performance of the resulting electron densities relative to an all-electron atomic density are given in the following subsection, followed by the application to experimental diffraction data sets from gold-, iodine- and mercury-containing molecules.

### 4.1. Comparison of ECP valence electron densities and all-electron valence densities

The electron densities obtained by a calculation on a level of theory of unrestricted PBE0/def2-TZVP for Rb, I, Au and Hg atoms in their respective ground states are shown in Fig. 2 ▸. Table S1 shows the size of the parameter vectors for the elements used, the minimum and maximum exponents in the vector **b**, and the minimum value of *t* obtained.

The plotted electron densities (Fig. 2 ▸) show how the application of the correction function can introduce nodal behaviour similar to an all-electron calculation. Once these correction parameters are determined, no significant additional time is required to calculate the form factors compared with the Hirshfeld atoms. However, the calculation of Hirshfeld atoms from all-electron wavefunctions takes longer because the radial grids around the atomic core have to be more precise, and therefore the Fourier transform has to be performed on more grid points.

In most cases, the major contribution to the overall timing of HAR will still be the quantum chemical calculation itself. The timings of refinements using both approaches are compared in Section 4.3.

### 4.2. Comparison of scattering factors derived from ECP wavefunctions and all-electron calculations

**Figure 3:**
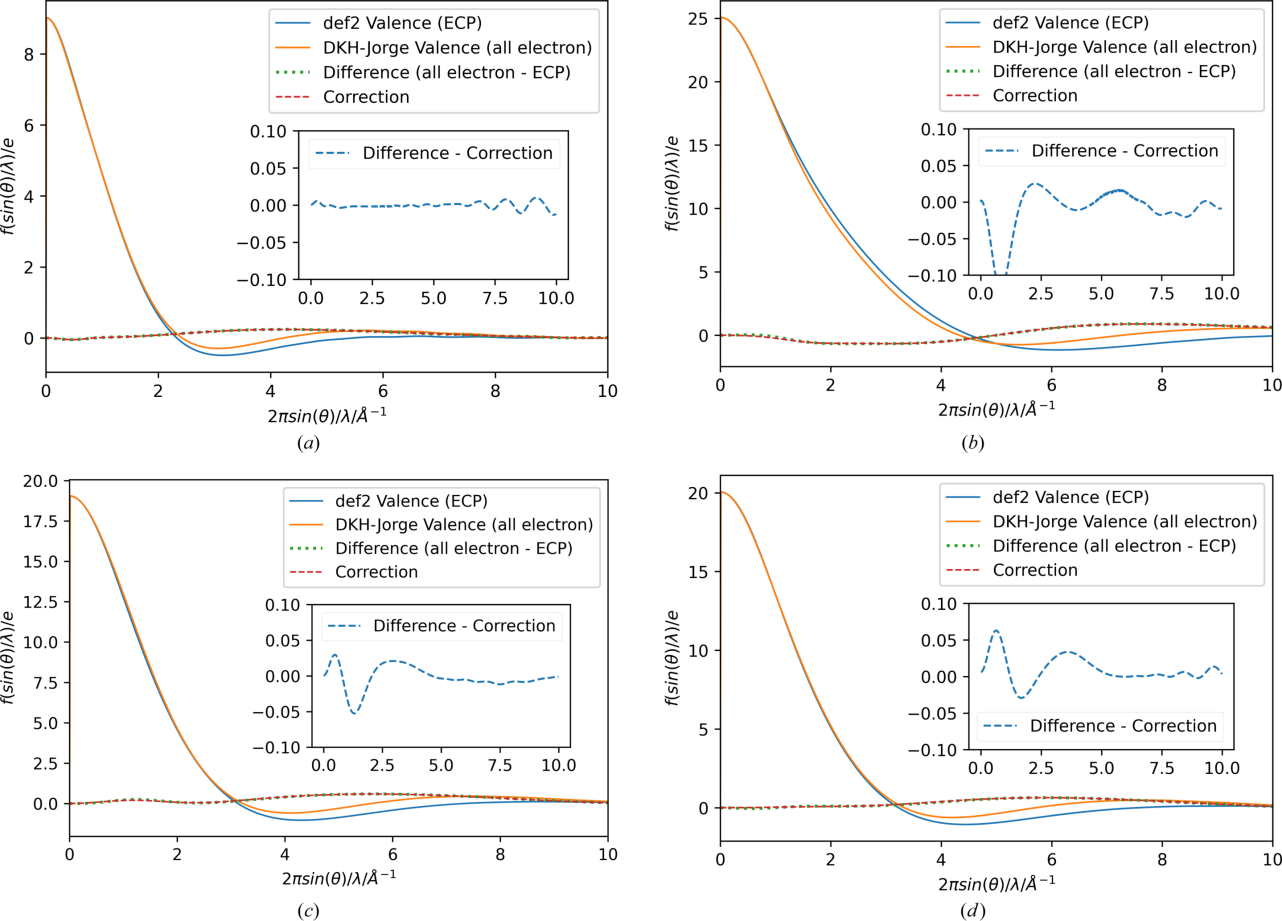
Plots of spherical scattering factor from the def2 wavefunction ρ_ECP_(*r*) (blue) and from the valence orbitals of the relativistic Jorge wavefunction ρ_val_(*r*) (orange), the difference between these scattering factors (green, dotted), and the fitted correction scattering factors obtained by the fitted density (red, dashed) for the elements (*a*) Rb, (*b*) I, (*c*) Au and (*d*) Hg. Insets contain the difference between the correction function scattering factors and those derived by the valence atoms to be fitted (blue, dashed).

The scattering factors were calculated using the same program as for the spherically averaged electron density, exchanging the function call for the electron density with that for the scattering factors and calculating the sets of spherically averaged valence scattering factors *f*_ECP_, *f*_val_ from the exact wavefunctions used for the electron density plots in Fig. 2 ▸. The resulting scattering factors were supplemented by the correction scattering factor applying equation (4) and are plotted in Fig. 3 ▸. The correction functions are almost indistinguishable from the difference between the all-electron calculation and the ECP-based results. This confirms that the fit was successful in real space and can compensate for the differences between the nodal behaviour of the calculated orbitals in reciprocal space. An enlarged plot of the difference between the target difference and the fit is shown in an inset for each plot.

**Figure 4:**
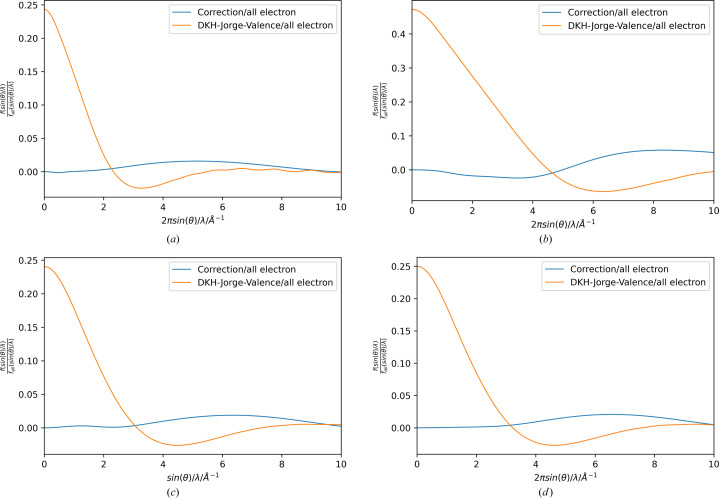
Plots of ratios between correction spherical scattering factor and all-electron scattering factor from the relativistic calculation (blue) and between the valence scattering factor and the all-electron scattering factors (orange) for the elements (*a*) Rb, (*b*) I, (*c*) Au and (*d*) Hg.

However, the differences between the two curves, as shown in the insets of Fig. 3 ▸, are relatively small compared with the absolute values of the valence scattering factors at low resolution (orange and blue), so the ratio between the correction function and the overall scattering factor, including all electrons of the wavefunction calculated using scalar relativistic methods, is plotted in Fig. 4 ▸. The relative contribution of the valence orbitals to the overall scattering factor is highest in the low-resolution region. In contrast, the contribution of the scattering factor obtained by the correction functions is highest at higher resolution. This observation is not surprising, given the reciprocal relationship between the expansion of the functions with the highest correction coefficients corresponding to the highest values of 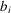, yielding the most contracted electron density closest to the atomic core. The same behaviour is observed for the scattering factors of the core orbitals, which have the most significant contribution relative to the highest-resolution reflections.

### 4.3. Comparison of refinement results and performance obtainedby ECP basis sets and all-electron relativistic *NoSpherA2*-HAR

To test the performance of the ECP basis in combination with the core densities obtained by the Slater-type densities and the calculated correction functions, a series of high-quality high-resolution data sets were collected on three compounds containing iodine, gold and mercury using Mo *K*α radiation: (dppm)Au_2_Br_2_ (McAuliffe *et al.*, 1979 ▸), [(OPPh_3_)_2_H][AuI_4_] and [(dppe)_2_Hg][PF_6_]_2_ [dppm is bis(diphenylphosphino)­methane, dppe is 1,2-bis(diphenylphosphino)ethane]. Details of their synthesis and crystallization are given in the supporting information. The data collection statistics for these data sets are summarized in Table 1 ▸. Refinements using both the ECP and the all-electron densities were performed with *NoSpherA2* (Kleemiss *et al.*, 2021 ▸) using r2SCAN (Furness *et al.*, 2020*a* ▸,*b* ▸) in all cases. No relativistic correction was included for the ECP-based calculations, and the def2-TZVP basis set (Weigend & Ahlrichs, 2005 ▸; Gulde *et al.*, 2012 ▸) was used. For the all-electron calculations, the Jorge-TZVP-DKH basis set (Campos & Jorge, 2013 ▸; Barros *et al.*, 2010 ▸; Machado *et al.*, 2009 ▸; Camiletti *et al.*, 2008 ▸; Canal Neto *et al.*, 2005 ▸; De Oliveira *et al.*, 2018 ▸; Martins *et al.*, 2016 ▸; Campos *et al.*, 2017 ▸) was chosen, in combination with the possible permutations of the relativistic approximations. All calculations were performed using *ORCA* Version 5.0.4 on a desktop computer with 12 cores on a Threadripper 5965WX with 256 GB of memory, with grid accuracy set to ‘Normal’ and the convergence set to ‘NormalConv’ without a solvation model. The required computation times using the different levels of theory and relativistic approximations are summarized in Table 2 ▸.

The speedup observed when using ECPs is most evident in the wavefunction calculation (def2-TZVP versus Jorge-TZVP-DKH in Table 2 ▸). Compared with DKH2-based calculations, significant speedup can also be observed when using ZORA or IORA. In the case of [(dppe)_2_Hg](PF_6_)_2,_ a speedup of a factor of 2.33 was observed. Even with only one atom bearing electrons modelled by an ECP, as in [(dppe)_2_Hg](PF_6_)_2_, an additional speedup of a factor of 1.62 can be observed when comparing ZORA and def2-ECP-based calculations. In the case of the gold-based compounds, the speedup between DKH2 and ZORA is less pronounced (factors of 1.63 and 1.14, respectively), but the presence of more atoms bearing ECPs leads to speedups from ZORA compared with ECPs with factors of 1.34 and 1.69, respectively.

The calculation of the scattering factors still requires elaborate numerical integration grids for ECPs – for Au/Hg, the atomic grids for all-electron calculations have 10580/11018 points; the def2-based grids only need 6262/6372 gridpoints – which reduces the time required for the observed cases by only a few milliseconds to seconds in the best case.

All refinements are compared in terms of the *R* values *R*_1_ and *wR*_2_, the highest and lowest residual density present in the unit cell, and *e*_gross_ as a measure of integrated residual density (Meindl & Henn, 2008 ▸) in Table 3 ▸. A visualization of the complete fractal dimension plots is shown in Figs. S1–S3. The refinements are nearly identical, with only the refinements of (dppm)Au_2_Br_2_ showing a slightly more symmetric distribution of the minimum and maximum residual density around 0. There is no systematic improvement or disadvantage to the use of ECPs in refining the systems studied.

## 5. Conclusion

The zeroth-order relativistic approximation is the best choice for the tested systems when using all-electron basis sets. While the difference from the results obtained by other all-electron methods is marginal, the improvement in timing is sometimes almost a factor of 2. Therefore, the default relativistic treatment for *ORCA* with all-electron basis sets in *NoSpherA2* was changed to ZORA and will be reported in the CIFs created automatically by *NoSpherA2*.

Our results clearly show that the refinement of structures containing heavy elements can benefit considerably from using ECPs in terms of computational effort required, without any significant loss of accuracy compared with results obtained after HAR using all-electron basis sets. The speedup depends on the number of heavy elements in the calculated wavefunction, so larger structures with multiple heavy element atoms benefit the most. Similar refinement statistics and residual density maps are obtained when comparing the results of the two approaches. Therefore, the use of ECPs is generally recommended, as it saves time and computational resources without compromising refinement accuracy or quality.

## Supplementary Material

Crystal structure: contains datablock(s) dppe2HgPF62_IAM, Ph3PO2HAuI4_IAM. DOI: 10.1107/S1600576725000901/woz5004sup1.cif

Additional information and figures, including fractal dimension plots and synthesis conditions, plots of density error for all elements, and deformation density comparisons between all-electron and ECP calculations. DOI: 10.1107/S1600576725000901/woz5004sup2.pdf

Raw data (including frames) of dppm(AuBr)2: https://doi.org/10.5281/zenodo.13789873

Raw data (including frames) of [(dppe)2Hg][PF6]2: https://doi.org/10.5281/zenodo.13790193

Raw data (including frames) of [(Ph3PO)2H][AuI4]: https://doi.org/10.5281/zenodo.13790014

ZIP archive including CIFs with processed intensities and calculated intensities, structural model and fit parameters, and tscb files required for reproduction of the refinement results: https://doi.org/10.5281/zenodo.13951060

CCDC references: 2386372, 2386374

## Figures and Tables

**Table 1:** Crystallographic data for (dppm)Au_2_Br_2_, [(OPPh_3_)_2_H][AuI_4_] and [(dppe)_2_Hg][PF_6_]_2_

Compound name	(dppm)Au_2_Br_2_	[(OPPh_3_)_2_H][AuI_4_]	[(dppe)_2_Hg][PF_6_]_2_
Sum formula	C_25_H_22_Au_2_Br_2_P_2_	C_36_H_30_AuI_4_O_2_P_2_	C_52_H_48_F_12_HgP_6_
Space group	*C*2/*c*	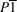	*P*1
*a* (Å)	31.0668 (7)	9.23767 (5)	9.9613 (1)
*b* (Å)	7.30892 (3)	10.52981 (5)	11.6741 (2)
*c* (Å)	18.3076 (4)	10.54412 (7)	11.9272 (2)
α (°)	90	102.0929 (5)	102.495 (1)
β (°)	142.559 (5)	112.8397 (6)	107.125 (1)
γ (°)	90	90.4679 (4)	96.849 (1)
*V* (Å^3^)	2527.2 (3)	919.677 (10)	1268.99 (4)
*Z*	4	1	1
*Z*′	0.5	1	1
No. of reflections measured	134667	323488	271959
No. of merged reflections	6793	30754	33734
*d*_min_ (Å)	0.58	0.50	0.54
〈*I*/σ(*I*)〉	61.6	57.3	43.0
*R*_int_ (%)	4.12	3.50	3.53
Average multiplicity	19.82	10.52	8.06
CCDC Refcode of IAM results	2385139	2386374	2386372

**Table 2:** Comparison of timings rounded to seconds (wavefunction calculation/partitioning) for different models of (dppm)Au_2_Br_2_, [(OPPh_3_)_2_H][AuI_4_] and [(dppe)_2_Hg][PF_6_]_2_

Level of theory	(dppm)Au_2_Br_2_	[(OPPh_3_)_2_H][AuI_4_]	[(dppe)_2_Hg](PF_6_)_2_
DKH2-r2SCAN/Jorge-TZVP-DKH	100/53	72/10	621/62
ZORA-r2SCAN/Jorge-TZVP-DKH	61/53	63/10	266/63
IORA-r2SCAN/Jorge-TZVP-DKH	96/52	122/10	318/63
r2SCAN/def2-TZVP	46/49	37/9	165/57
No. of atoms with ECPs	2	5	1

**Table 3:** Comparison of residual density, *R* values and *e*_gross_ refinement results

Level of theory	Min/max residual density†	*e*_gross_ ‡	*R*_1_/*wR*_2_
[(dppe)_2_Hg][PF_6_]_2_			
DKH2-r2SCAN/Jorge-TZVP-DKH	−1.3752/1.2489	28.9306	0.0171/0.0312
ZORA-r2SCAN/Jorge-TZVP-DKH	−1.3773/1.2495	28.9363	0.0171/0.0312
IORA-r2SCAN/Jorge-TZVP-DKH	−1.3816/1.2535	28.8395	0.0171/0.0316
r2SCAN/def2-TZVP	−1.3830/1.2588	28.9110	0.0171/0.0311
[(OPPh_3_)_2_H][AuI_4_]			
DKH2-r2SCAN/Jorge-TZVP-DKH	−1.6629/3.1812	37.0742	0.0256/0.0662
ZORA-r2SCAN/Jorge-TZVP-DKH	−1.6757/3.1850	37.0802	0.0256/0.0657
IORA-r2SCAN/Jorge-TZVP-DKH	−1.6602/3.1779	37.0491	0.0256/0.0659
r2SCAN/def2-TZVP	−1.6668/3.1943	37.1950	0.0256/0.0663
(dppm)Au_2_Br_2_			
DKH2-r2SCAN/Jorge-TZVP-DKH	−0.8233/0.9923	143.3873	0.0165/0.0258
ZORA-r2SCAN/Jorge-TZVP-DKH	−0.8191/0.9974	143.3642	0.0165/0.0258
IORA-r2SCAN/Jorge-TZVP-DKH	−0.8381/0.9741	143.0681	0.0165/0.0257
r2SCAN/def2-TZVP	−0.8593/0.9586	143.1639	0.0165/0.0256

† Residual densities were obtained using a grid point separation of 0.1 Å rather than the default grids after refinements in *Olex2* to get a more detailed evaluation of residual density values.

‡ According to the definition of Meindl & Henn (2008 ▸).
