# Supplementary material for: The use of effective core potentials in Hirshfeld atom refinement: making quantum crystallography faster in NoSpherA2
Source: J Appl Crystallogr. 2025 Mar 7;58(Pt 2):374–82. doi: 10.1107/S1600576725000901 (PMC11957402; doi:10.1107/S1600576725000901)
Supplement: Supplementary file 2 [file j-58-00374-sup2.pdf]

JOURNAL OF
APPLIED
CRYSTALLOGRAPHY

Volume 58 (2025)

Supporting information for article:

The use of effective core potentials in Hirshfeld atom refinement - making quantum crystallography faster in *NoSpherA2*

Florian Kleemiss, Florian Meurer, Ilya G. Shenderovich and Michael Bodensteiner

S1. Table of Gaussian correction function parameters

The choice of relativistic treatment for the ORCA program package is controlled by the structure variable snum.NoSpherA2.ORCA_Relativistic which can be changed using the „spy.editparams(snum.NoSpherA2.ORCA_Relativistic)“ instruction inside the terminal of Olex2.

Table S1: Overview of parameters for correction functions $\Delta\rho(\boldsymbol{a},\boldsymbol{b},r)$ obtained after minimization of objective function t.

Element	Number of radial Gaussian functions	Highest b	Lowest b	Integral of $\Delta\rho(\boldsymbol{a},\boldsymbol{b},r)$	Achieved error t in %
Rb	15	2662141.6	0.03128	3.3586E-05	0.100
Sr	18	2719103.4	0.19023	1.8401E-03	0.799
Y	26	894012.6	0.03731	1.1934E-05	0.834
Zr	18	23546285.4	0.06156	5.2255E-04	0.613
Nb	23	6200938.4	0.09455	-5.1097E-05	0.554
Mo	19	77331979.8	0.03806	-8.8913E-05	0.675
Tc	17	13173640.9	0.14557	-1.2575E-04	0.156
Ru	26	6200771.2	0.12363	-3.7076E-05	0.708
Rh	27	6200854.7	0.10445	5.4025E-06	0.108
Pd	18	21137313.3	0.07519	-3.4368E-04	0.122
Ag	23	6200907.0	0.30537	1.4732E-04	0.842
Cd	23	6200811.1	0.15026	-2.0588E-05	0.619
In	17	6200806.4	1.44693	8.2154E-05	0.771
Sn	26	6200800.2	0.01879	-3.9882E-06	0.399
Sb	26	6200887.1	0.25129	6.3728E-05	0.241
Te	22	6200844.4	0.59637	1.1332E-04	0.857
I	21	6200857.9	0.12625	-6.3464E-07	0.506
Xe	18	14336173.5	0.03998	2.8969E-04	0.169
Cs	15	4292242.5	0.01504	-4.7114E-05	0.097
Ba	15	3418974.6	0.02625	-3.2615E-04	0.828
La	15	96986948.6	1.24797	3.3405E-03	0.922
Ce	17	9790439.6	0.03879	6.0713E-05	0.560
Pr	20	11902889.7	1.11843	7.6664E-04	0.894
Nd	17	12944205.4	0.41140	-1.7705E-04	0.751
Pm	18	10498523.2	2.24234	2.7906E-04	0.557
Sm	14	7379525.0	0.07058	-1.8808E-06	0.163
Eu	28	6201080.4	0.02975	-1.1572E-04	0.946
Gd	16	6204435.6	0.08871	-2.5756E-04	0.847
Tb	24	6828952.9	0.31593	-2.4959E-04	0.778
Dy	21	6200944.6	0.02343	1.5188E-05	0.672
Ho	15	9616923.1	0.33215	5.5617E-05	0.490

Er	28	6200898.8	0.06248	1.8386E-05	0.130
Tm	17	8857004.7	0.23152	4.1184E-04	0.375
Yb	28	6200720.7	0.38518	-1.5085E-04	0.774
Lu	17	14422483.0	0.81269	-3.7970E-04	0.856
Hf	16	30491740.3	0.08254	1.2475E-05	0.330
Ta	24	6200855.0	0.06153	-8.8657E-06	0.775
W	22	24098607.3	0.09459	6.6172E-06	0.775
Re	15	27056655.2	0.03285	1.1387E-04	0.099
Os	14	915498921.9	0.05732	-6.9712E-05	0.833
Ir	14	15286342.7	0.09342	1.3586E-04	0.820
Pt	21	342134486.4	0.02870	4.5403E-04	0.726
Au	21	23161157.9	0.36624	4.5705E-05	0.651
Hg	19	23738710.1	0.14601	4.9640E-04	0.162
Tl	26	22825551.5	0.04267	6.1703E-05	0.662
Pb	15	20080286.4	0.04438	-3.5054E-05	0.369
Bi	12	31087737.4	1.97683	1.4923E-04	0.531
Po	23	9999814.1	0.54536	-8.0462E-05	0.701
At	13	44580957.6	2.33567	3.1665E-04	0.677
Rn	20	9200810.9	0.98596	-1.2805E-04	0.854

S2. Fractal Dimension Plots of refinements

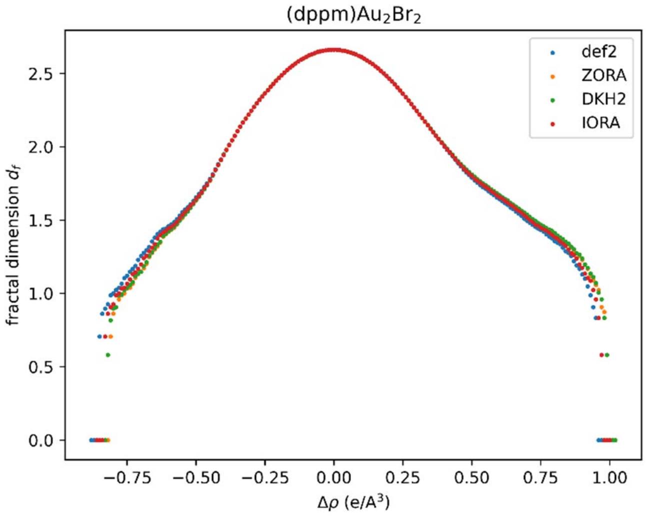

Figure S1 Fractal dimension plot after refinement of (dppm)Au_2Br_2 using def2-TZVP (with ECP, blue) or Jorge-TZVP-DKH with ZORA(yellow), DKH (green) and IORA (red) relativistic approximation.

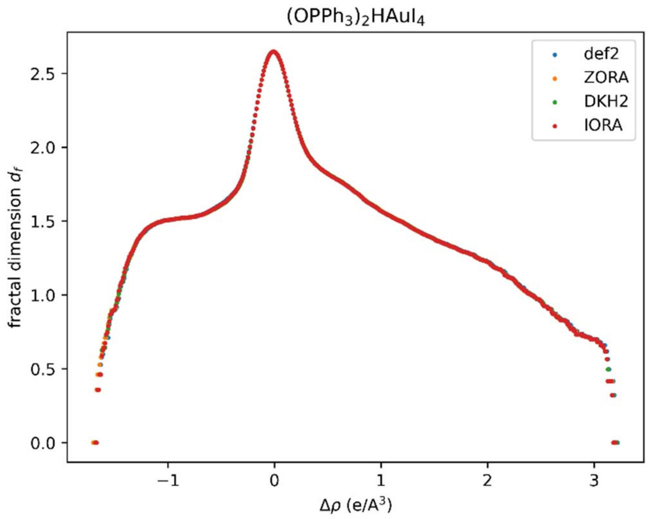

Figure S2 Fractal dimension plot after refinement of $(OPPh_3)_2HAuI_4$ using def2-TZVP (with ECP, blue) or Jorge-TZVP-DKH with ZORA(yellow), DKH (green) and IORA (red) relativistic approximation.

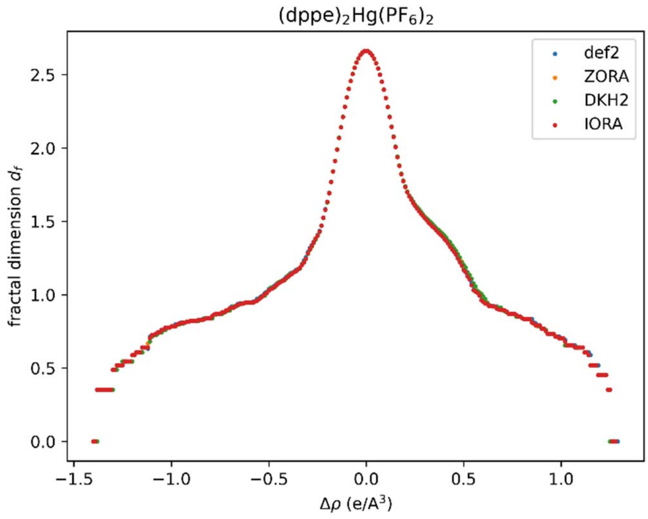

Figure S3 Fractal dimension plot after refinement of $(dppe)_2Hg(PF_6)_2$ using def2-TZVP (with ECP, blue) or Jorge-TZVP-DKH with ZORA(yellow), DKH (green) and IORA (red) relativistic approximation.

S3. Synthesis and Crystallization of Compounds investigated

S3.1. DPPM(AuBr)$_2$

DPPM(AuBr)$_2$ was synthesized according to the literature by McAuliffe et al. (C. A. McAuliffe , R. V. Parish and P. D. Randall , *Dalton Trans.*, 1979, 1730, https://doi.org/10.1039/DT9790001730). Crystals suitable for X-ray crystallography were obtained by solvent evaporation of a solution of DPPM(AuBr)$_2$ in dichloromethane.

The refinement results obtained by spherical atom modelling was uploaded to the CCDC with reference number 2385139.
Full raw (frames and metadata) and processed data used for the refinements are available at: https://doi.org/10.5281/zenodo.13789873

S3.2. [(DPPE)$_2$Hg][PF$_6$]$_2$

[(DPPE)$_2$Hg][PF$_6$]$_2$ was prepared by dissolving 400 mg of ethane,1,2-diyl)bis(diphenylphosphane) (DPPE) together with 100 mg of PF_6 in 50 ml dimethylsulfoxide. The solution was heated to a temperature of 50°C, where 250 mg of mercury(I)nitrate was added. The mixture was left stirring overnight. Upon the addition of an excess amount of water, large white flakes precipitated. The precipitate was filtered and washed with chloroform. Crystals suitable for X-ray diffraction were obtained by solvent diffusion of hexane layered on a solution of [(DPPE)$_2$Hg][PF$_6$]$_2$ in dichloromethane after several days. The refinement results obtained by spherical atom modellin was uploaded to the CCDC with reference number 2386374.
Full raw (frames and metadata) and processed data used for the refinements are available at: https://doi.org/10.5281/zenodo.13790193

S3.3. [(Ph$_3$PO)$_2$H][AuI$_4$]

Similar cation structures: IZOVAU, IZOVEY, PORNUF, RIDMAT, TPOPH, TPOPHX01
A solution of $HAuCl_4$ in ethanol was mixed with two equivalents of PPh_3 and left stirring for three hours at 50°C. The formed [(Ph$_3$PO)$_2$H][AuCl$_4$] was dissolved in 50 mL dichloromethane and added to 50 mL excess of KI in water, then stirred for two hours. After separating the water and DCM phase, suitable crystals for X-ray diffraction were obtained from slow evaporation of the dichloromethane solvent.
The refinement results obtained by spherical atom modellin was uploaded to the CCDC with reference number 2386372.
Full raw (frames and metadata) and processed data used for the refinements are available at: https://doi.org/10.5281/zenodo.13790014

S4. Plots of Radial Densities of Elements with ECPs in def2-family basis sets

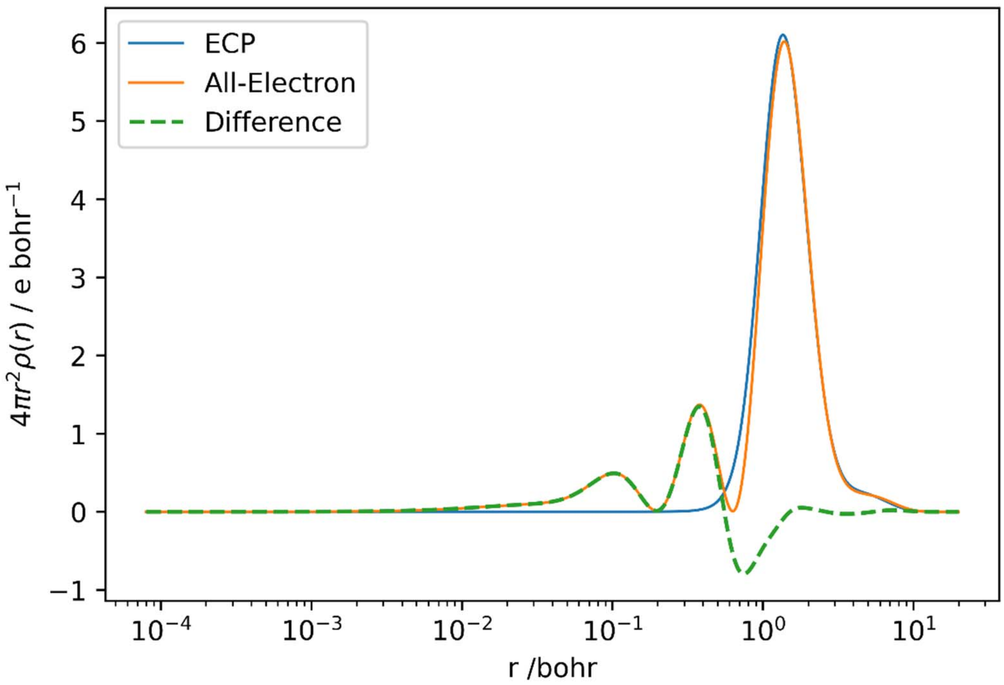

Figure S4 Difference in radial electron distribution function of 3s, 3p, 3d, 4s, 4p and 5s valence orbital for Rb using def2-TZVPP (Leininger *et al.*, 1996; Peterson *et al.*, 2003; Weigend & Ahlrichs, 2005; Gulde *et al.*, 2012; Dolg *et al.*, 1989; Andrae *et al.*, 1990) (blue), using an all-electron relativistic Jorge-TZVP-DKH calculation (orange) and the difference between the two distributions (green, dashed) against distance from the nucleus on a logarithmic scale.

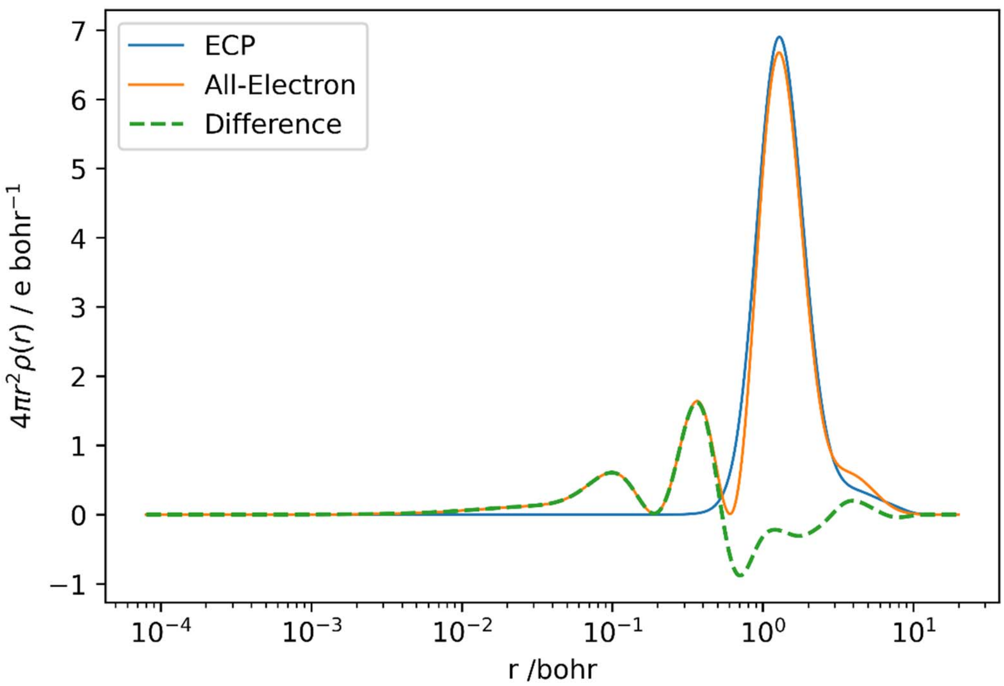

Figure S5 Difference in radial electron distribution function of 3s, 3p, 3d, 4s, 4p and 5s valence orbital for Sr using def2-TZVPP (Leininger *et al.*, 1996; Peterson *et al.*, 2003; Weigend & Ahlrichs, 2005; Gulde *et al.*, 2012; Dolg *et al.*, 1989; Andrae *et al.*, 1990) (blue), using an all-electron relativistic Jorge-TZVP-DKH calculation (orange) and the difference between the two distributions (green, dashed) against distance from the nucleus on a logarithmic scale.

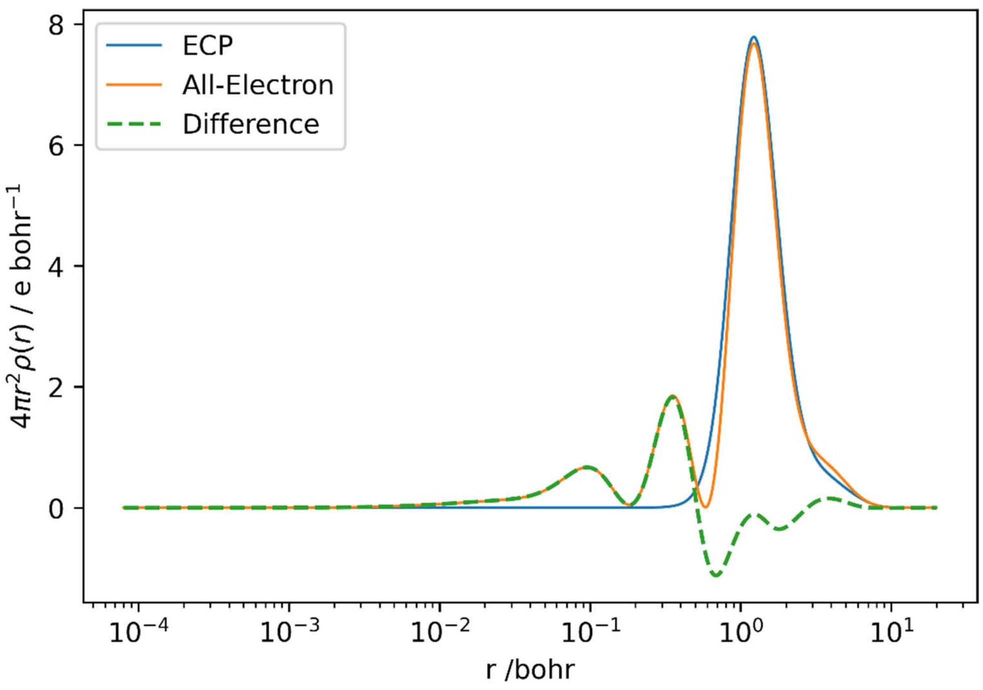

Figure S6 Difference in radial electron distribution function of 3s, 3p, 3d, 4s, 4p, 4d and 5s valence orbital for Y using def2-TZVPP (Leininger *et al.*, 1996; Peterson *et al.*, 2003; Weigend & Ahlrichs, 2005; Gulde *et al.*, 2012; Dolg *et al.*, 1989; Andrae *et al.*, 1990) (blue), using an all-electron relativistic Jorge-TZVP-DKH calculation (orange) and the difference between the two distributions (green, dashed) against distance from the nucleus on a logarithmic scale.

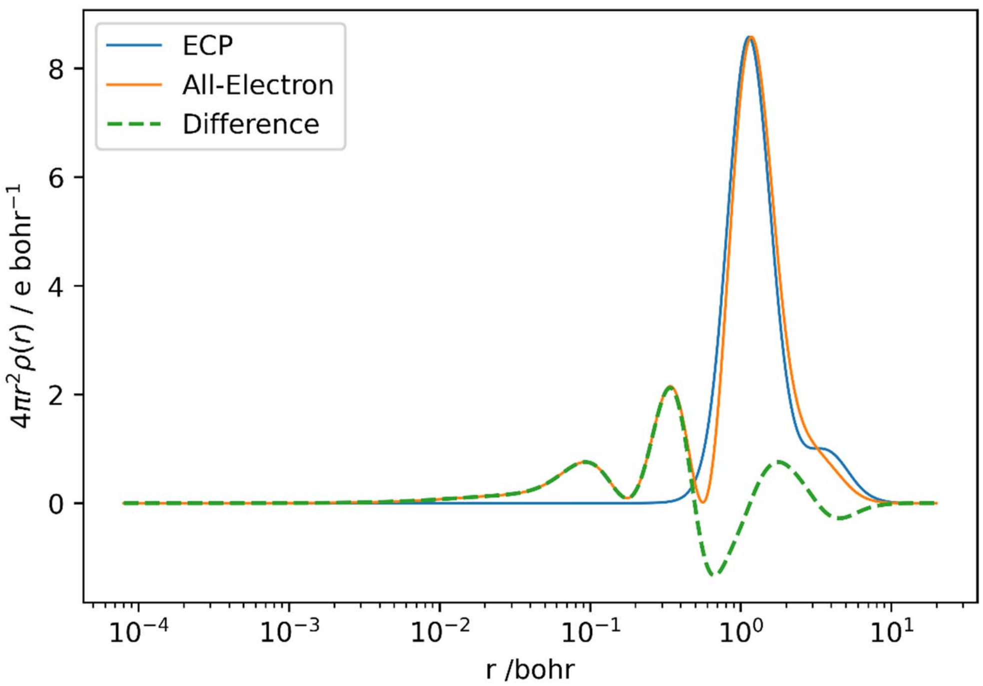

Figure S7 Difference in radial electron distribution function of 3s, 3p, 3d, 4s, 4p, 4d and 5s valence orbital for Zr using def2-TZVPP (Leininger *et al.*, 1996; Peterson *et al.*, 2003; Weigend & Ahlrichs, 2005; Gulde *et al.*, 2012; Dolg *et al.*, 1989; Andrae *et al.*, 1990) (blue), using an all-electron relativistic Jorge-TZVP-DKH calculation (orange) and the difference between the two distributions (green, dashed) against distance from the nucleus on a logarithmic scale.

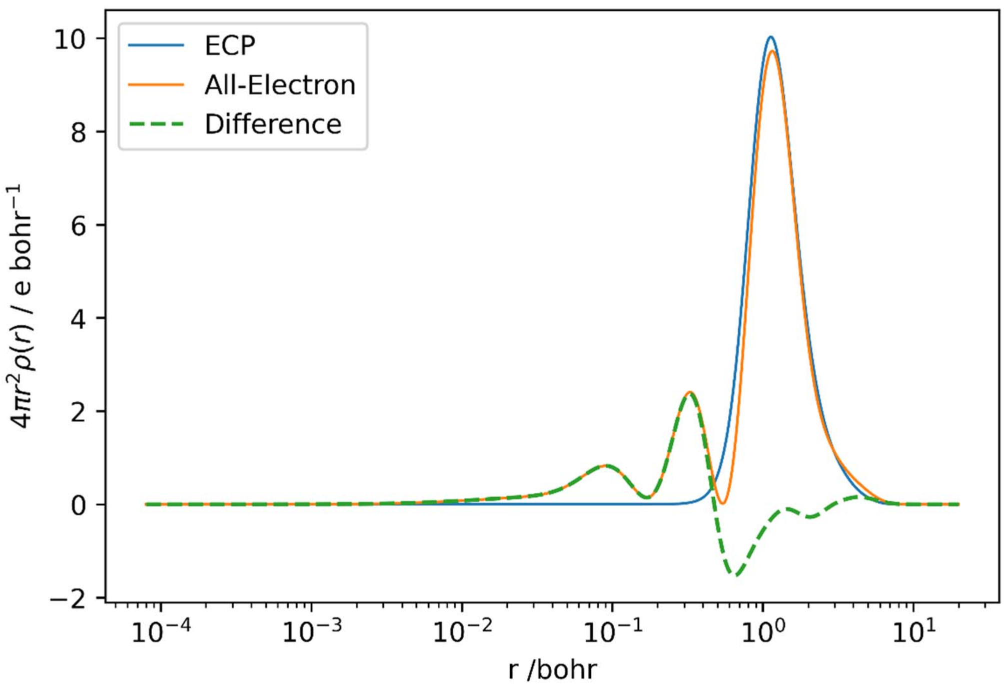

Figure S8 Difference in radial electron distribution function of 3s, 3p, 3d, 4s, 4p, 4d and 5s valence orbital for Nb using def2-TZVPP (Leininger *et al.*, 1996; Peterson *et al.*, 2003; Weigend & Ahlrichs, 2005; Gulde *et al.*, 2012; Dolg *et al.*, 1989; Andrae *et al.*, 1990) (blue), using an all-electron relativistic Jorge-TZVP-DKH calculation (orange) and the difference between the two distributions (green, dashed) against distance from the nucleus on a logarithmic scale.

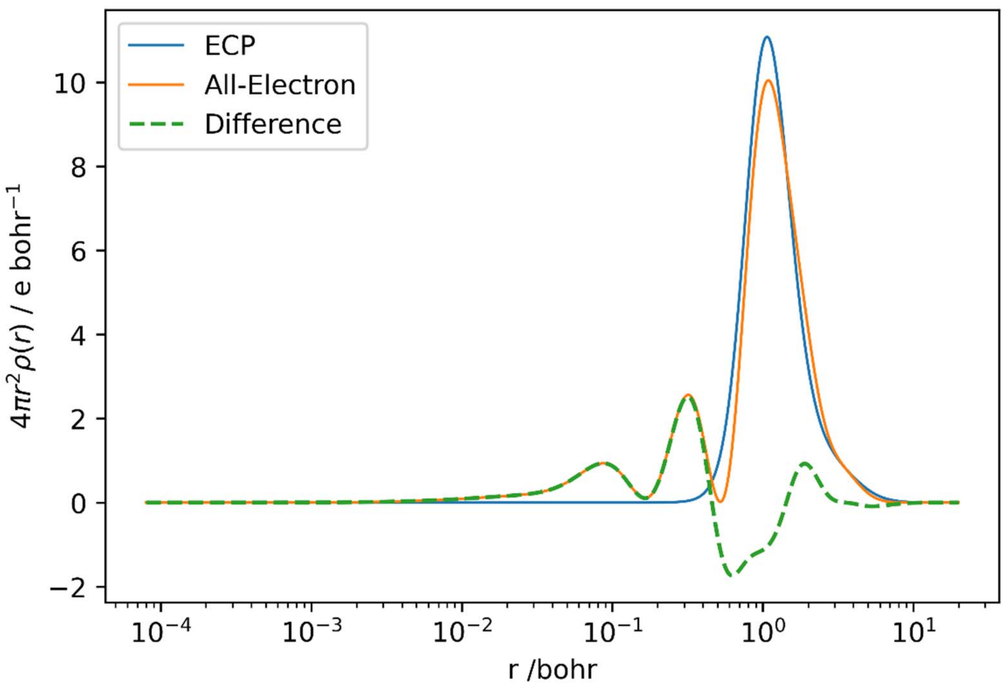

Figure S9 Difference in radial electron distribution function of 3s, 3p, 3d, 4s, 4p, 4d and 5s valence orbital for Mo using def2-TZVPP (Leininger *et al.*, 1996; Peterson *et al.*, 2003; Weigend & Ahlrichs, 2005; Gulde *et al.*, 2012; Dolg *et al.*, 1989; Andrae *et al.*, 1990) (blue), using an all-electron relativistic Jorge-TZVP-DKH calculation (orange) and the difference between the two distributions (green, dashed) against distance from the nucleus on a logarithmic scale.

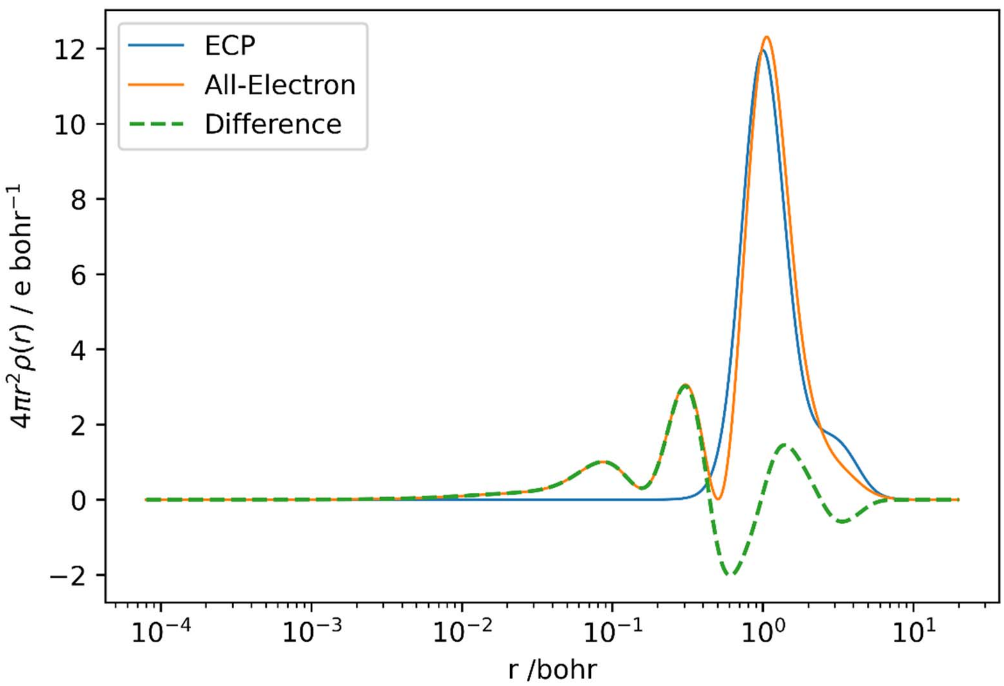

Figure S10 Difference in radial electron distribution function of 3s, 3p, 3d, 4s, 4p, 4d and 5s valence orbital for Tc using def2-TZVPP (Leininger *et al.*, 1996; Peterson *et al.*, 2003; Weigend & Ahlrichs, 2005; Gulde *et al.*, 2012; Dolg *et al.*, 1989; Andrae *et al.*, 1990) (blue), using an all-electron relativistic Jorge-TZVP-DKH calculation (orange) and the difference between the two distributions (green, dashed) against distance from the nucleus on a logarithmic scale.

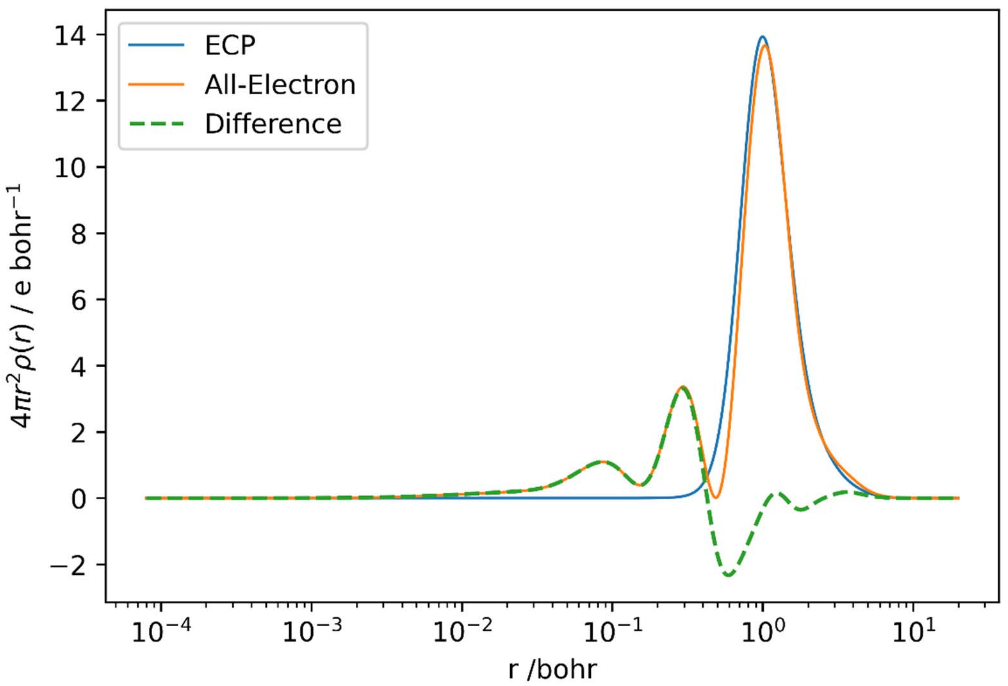

Figure S11 Difference in radial electron distribution function of 3s, 3p, 3d, 4s, 4p, 4d and 5s valence orbital for Ru using def2-TZVPP (Leininger *et al.*, 1996; Peterson *et al.*, 2003; Weigend & Ahlrichs, 2005; Gulde *et al.*, 2012; Dolg *et al.*, 1989; Andrae *et al.*, 1990) (blue), using an all-electron relativistic Jorge-TZVP-DKH calculation (orange) and the difference between the two distributions (green, dashed) against distance from the nucleus on a logarithmic scale.

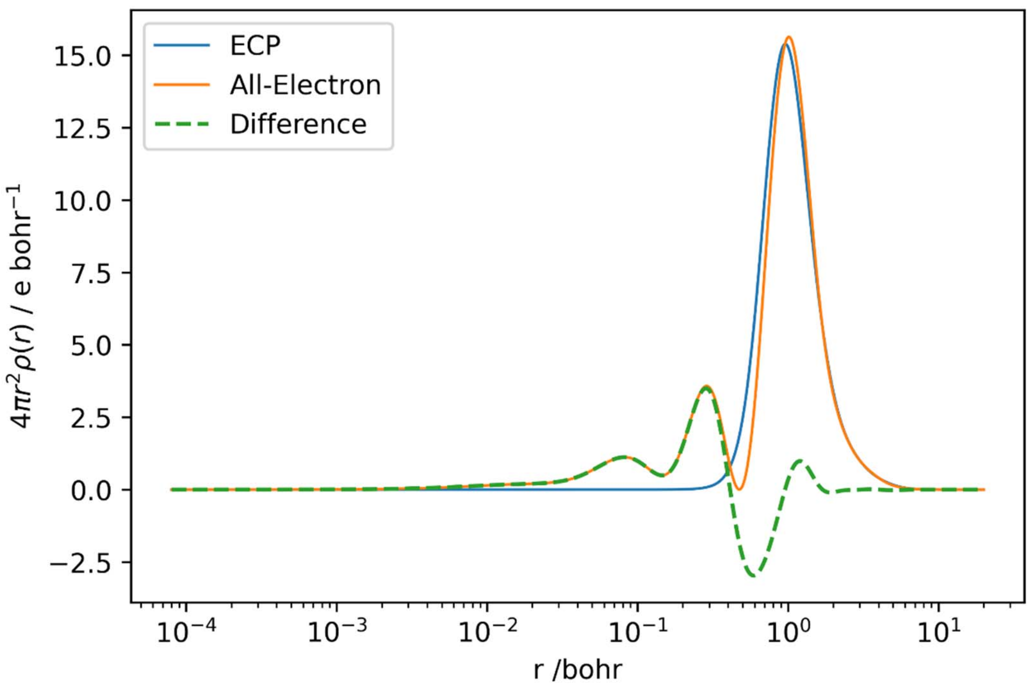

Figure S12 Difference in radial electron distribution function of 3s, 3p, 3d, 4s, 4p, 4d and 5s valence orbital for Rh using def2-TZVPP (Leininger *et al.*, 1996; Peterson *et al.*, 2003; Weigend & Ahlrichs, 2005; Gulde *et al.*, 2012; Dolg *et al.*, 1989; Andrae *et al.*, 1990) (blue), using an all-electron relativistic Jorge-TZVP-DKH calculation (orange) and the difference between the two distributions (green, dashed) against distance from the nucleus on a logarithmic scale.

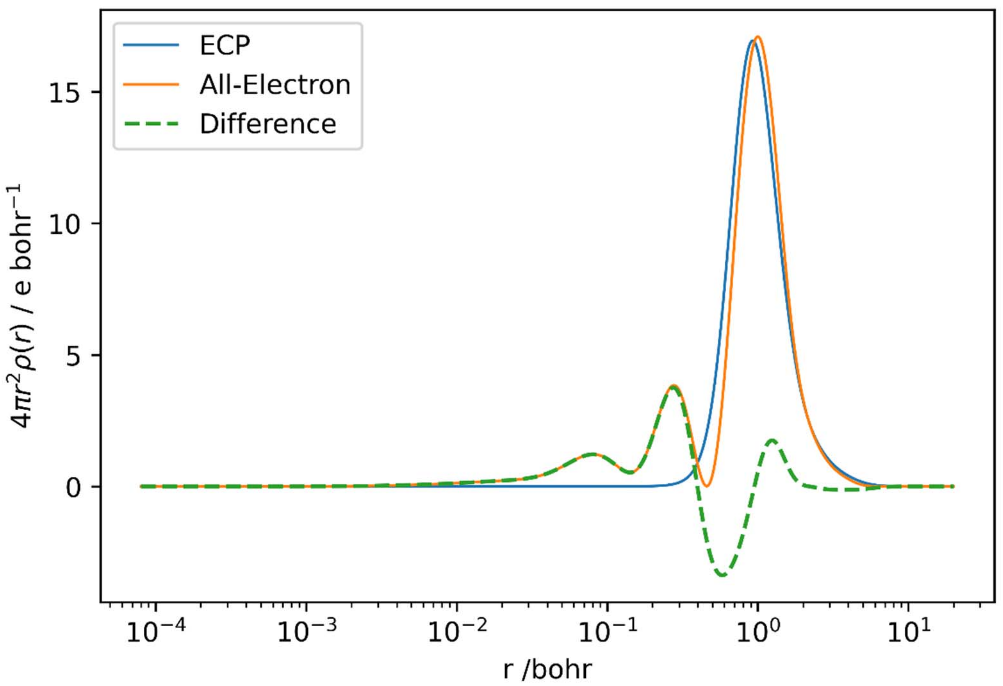

Figure S13 Difference in radial electron distribution function of 3s, 3p, 3d, 4s, 4p, 4d and 5s valence orbital for Pd using def2-TZVPP (Leininger *et al.*, 1996; Peterson *et al.*, 2003; Weigend & Ahlrichs, 2005; Gulde *et al.*, 2012; Dolg *et al.*, 1989; Andrae *et al.*, 1990) (blue), using an all-electron relativistic Jorge-TZVP-DKH calculation (orange) and the difference between the two distributions (green, dashed) against distance from the nucleus on a logarithmic scale.

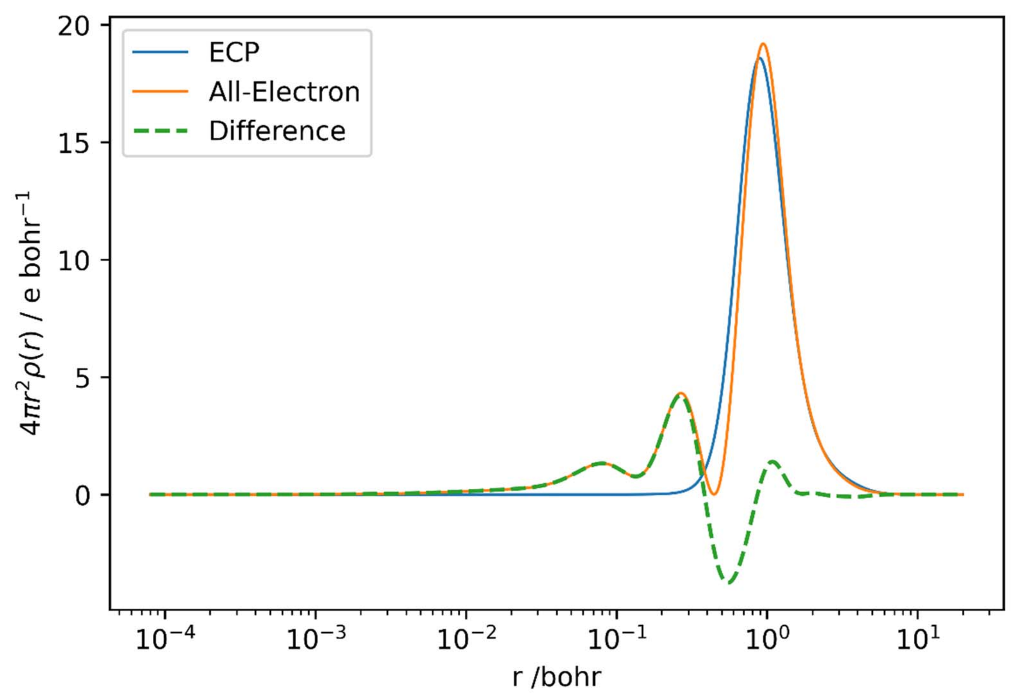

Figure S14 Difference in radial electron distribution function of 3s, 3p, 3d, 4s, 4p, 4d and 5s valence orbital for Ag using def2-TZVPP (Leininger *et al.*, 1996; Peterson *et al.*, 2003; Weigend & Ahlrichs, 2005; Gulde *et al.*, 2012; Dolg *et al.*, 1989; Andrae *et al.*, 1990) (blue), using an all-electron relativistic Jorge-TZVP-DKH calculation (orange) and the difference between the two distributions (green, dashed) against distance from the nucleus on a logarithmic scale.

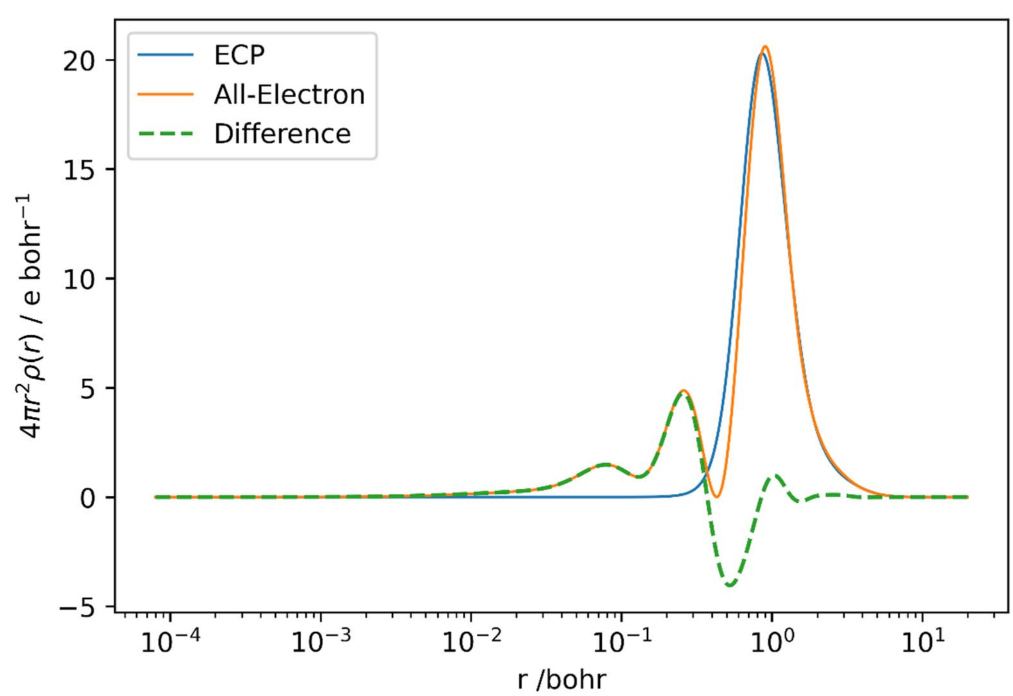

Figure S15 Difference in radial electron distribution function of 3s, 3p, 3d, 4s, 4p, 4d and 5s valence orbital for Cd using def2-TZVPP (Leininger *et al.*, 1996; Peterson *et al.*, 2003; Weigend & Ahlrichs, 2005; Gulde *et al.*, 2012; Dolg *et al.*, 1989; Andrae *et al.*, 1990) (blue), using an all-electron relativistic Jorge-TZVP-DKH calculation (orange) and the difference between the two distributions (green, dashed) against distance from the nucleus on a logarithmic scale.

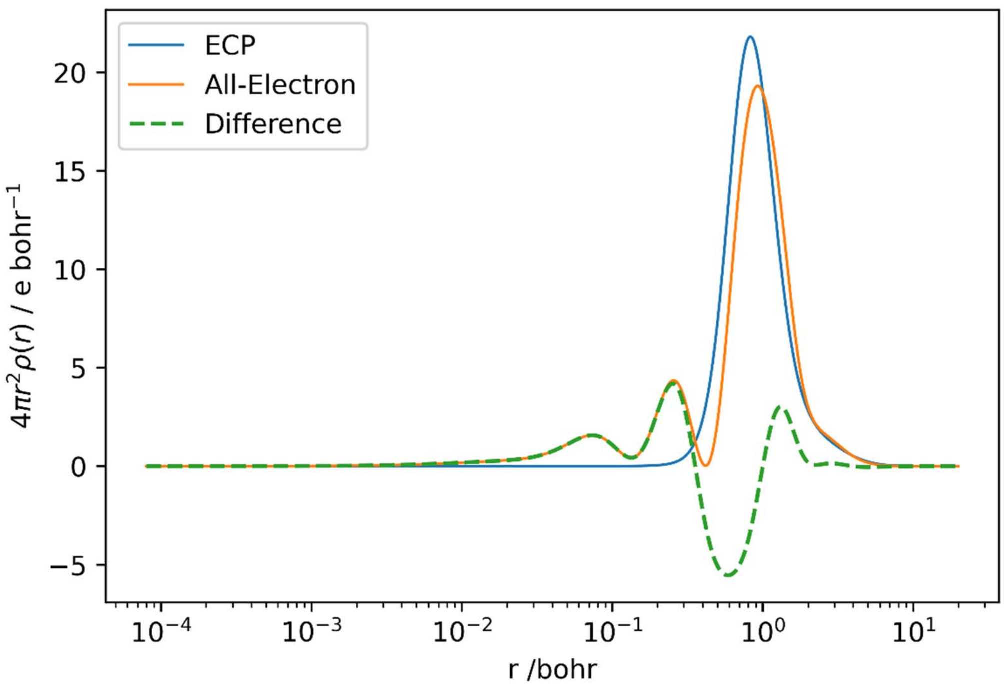

Figure S16 Difference in radial electron distribution function of 3s, 3p, 3d, 4s, 4p, 4d, 5s and 5ds valence orbital for In using def2-TZVPP (Leininger *et al.*, 1996; Peterson *et al.*, 2003; Weigend & Ahlrichs, 2005; Gulde *et al.*, 2012; Dolg *et al.*, 1989; Andrae *et al.*, 1990) (blue), using an all-electron relativistic Jorge-TZVP-DKH calculation (orange) and the difference between the two distributions (green, dashed) against distance from the nucleus on a logarithmic scale.

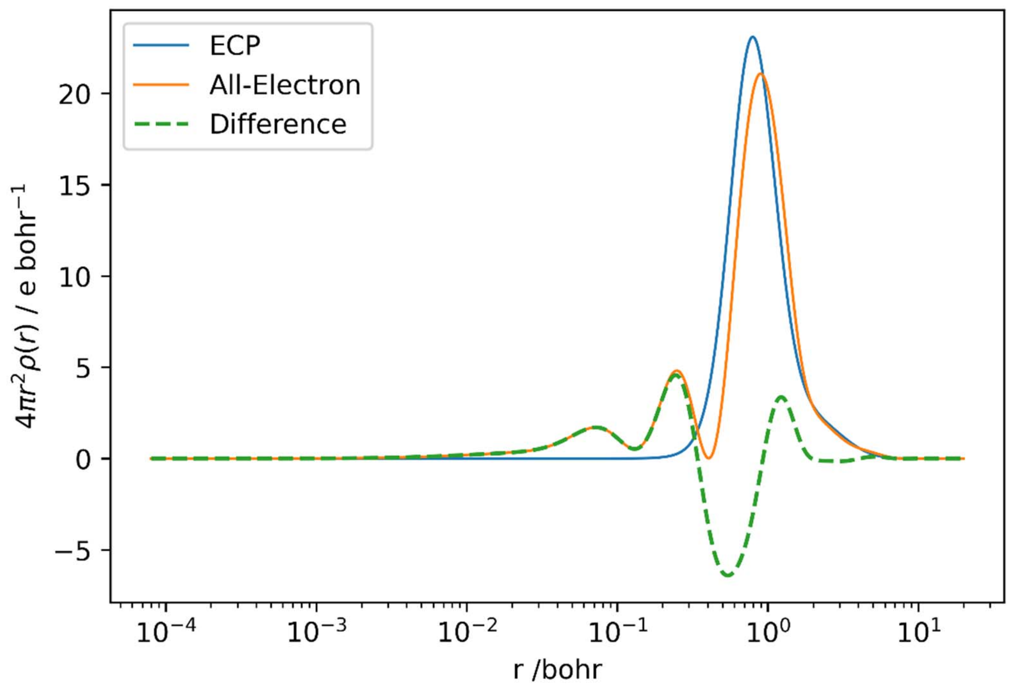

Figure S17 Difference in radial electron distribution function of 3s, 3p, 3d, 4s, 4p, 4d, 5s and 5p valence orbital for Sn using def2-TZVPP (Leininger *et al.*, 1996; Peterson *et al.*, 2003; Weigend & Ahlrichs, 2005; Gulde *et al.*, 2012; Dolg *et al.*, 1989; Andrae *et al.*, 1990) (blue), using an all-electron relativistic Jorge-TZVP-DKH calculation (orange) and the difference between the two distributions (green, dashed) against distance from the nucleus on a logarithmic scale.

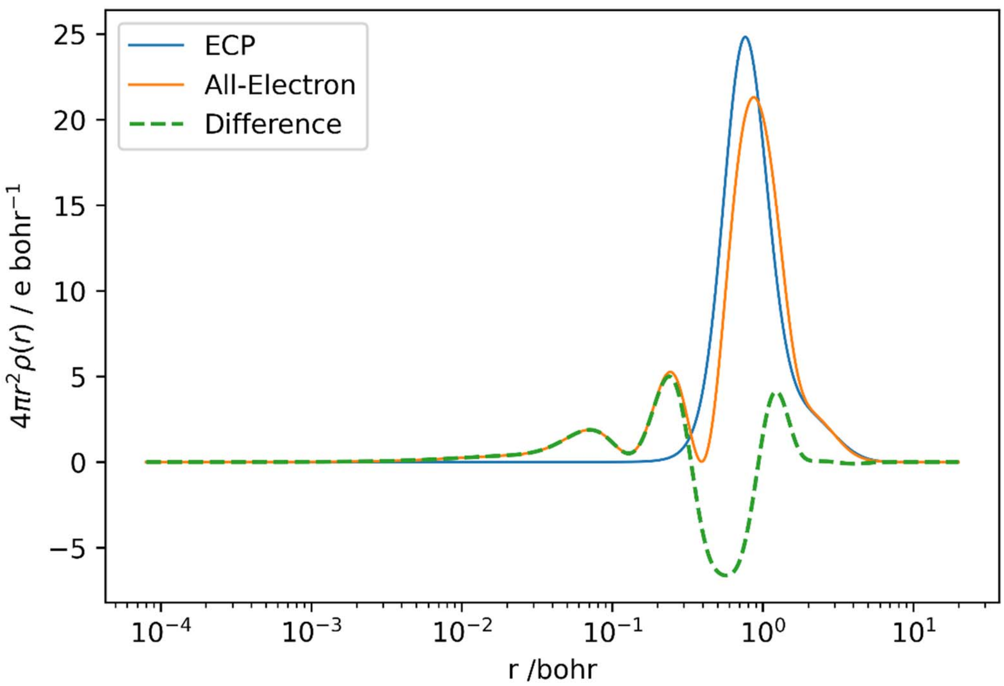

Figure S18 Difference in radial electron distribution function of 3s, 3p, 3d, 4s, 4p, 4d, 5s and 5p valence orbital for Sb using def2-TZVPP (Leininger *et al.*, 1996; Peterson *et al.*, 2003; Weigend & Ahlrichs, 2005; Gulde *et al.*, 2012; Dolg *et al.*, 1989; Andrae *et al.*, 1990) (blue), using an all-electron relativistic Jorge-TZVP-DKH calculation (orange) and the difference between the two distributions (green, dashed) against distance from the nucleus on a logarithmic scale.

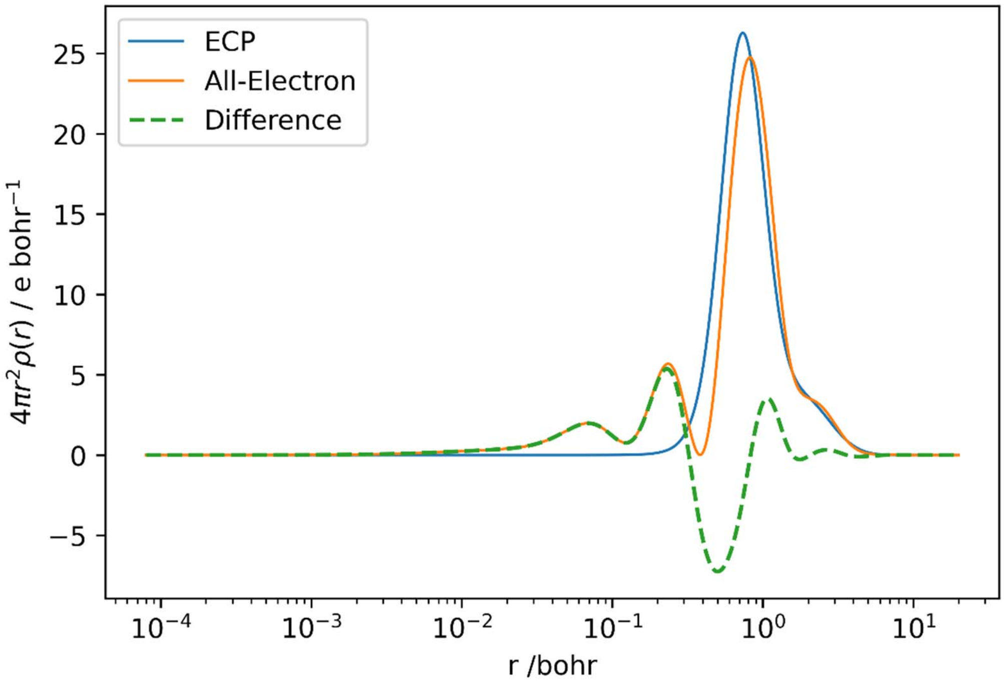

Figure S19 Difference in radial electron distribution function of 3s, 3p, 3d, 4s, 4p, 4d, 5s and 5p valence orbital for Te using def2-TZVPP (Leininger *et al.*, 1996; Peterson *et al.*, 2003; Weigend & Ahlrichs, 2005; Gulde *et al.*, 2012; Dolg *et al.*, 1989; Andrae *et al.*, 1990) (blue), using an all-electron relativistic Jorge-TZVP-DKH calculation (orange) and the difference between the two distributions (green, dashed) against distance from the nucleus on a logarithmic scale.

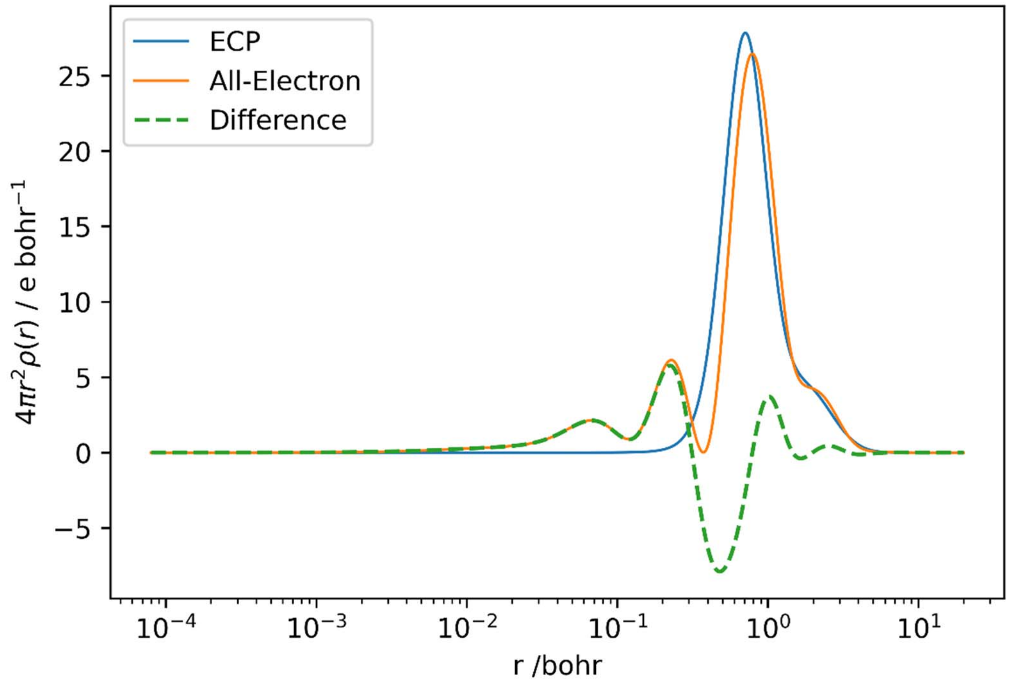

Figure S20 Difference in radial electron distribution function of 3s, 3p, 3d, 4s, 4p, 4d, 5s and 5p valence orbital for I using def2-TZVPP (Leininger *et al.*, 1996; Peterson *et al.*, 2003; Weigend & Ahlrichs, 2005; Gulde *et al.*, 2012; Dolg *et al.*, 1989; Andrae *et al.*, 1990) (blue), using an all-electron relativistic Jorge-TZVP-DKH calculation (orange) and the difference between the two distributions (green, dashed) against distance from the nucleus on a logarithmic scale.

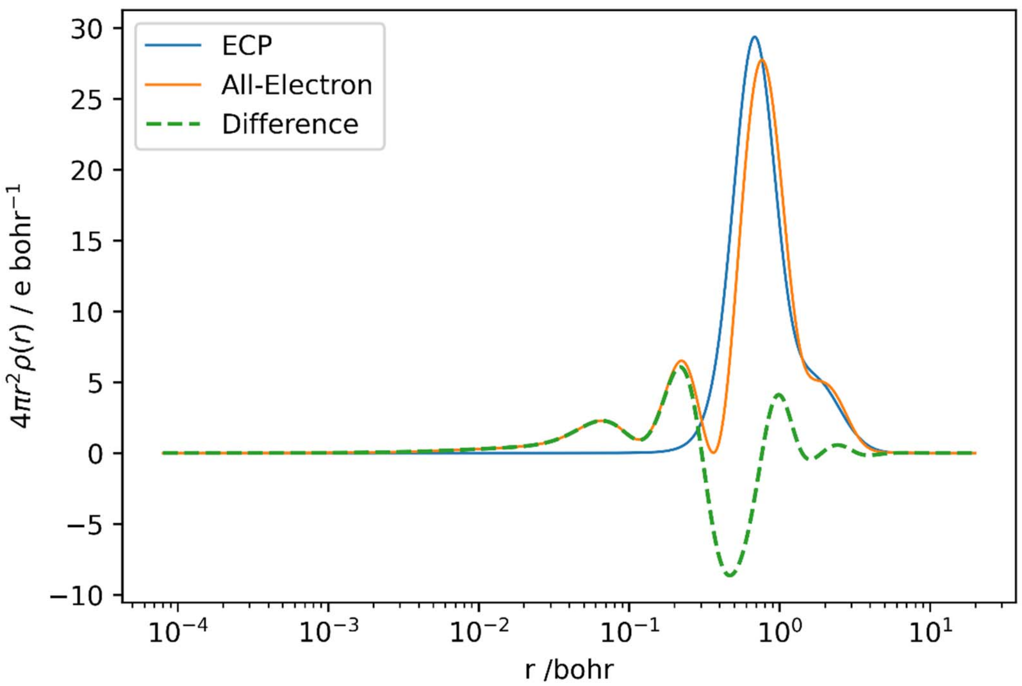

Figure S21 Difference in radial electron distribution function of 3s, 3p, 3d, 4s, 4p, 4d, 5s and 5p valence orbital for Xe using def2-TZVPP (Leininger *et al.*, 1996; Peterson *et al.*, 2003; Weigend & Ahlrichs, 2005; Gulde *et al.*, 2012; Dolg *et al.*, 1989; Andrae *et al.*, 1990) (blue), using an all-electron relativistic Jorge-TZVP-DKH calculation (orange) and the difference between the two distributions (green, dashed) against distance from the nucleus on a logarithmic scale.

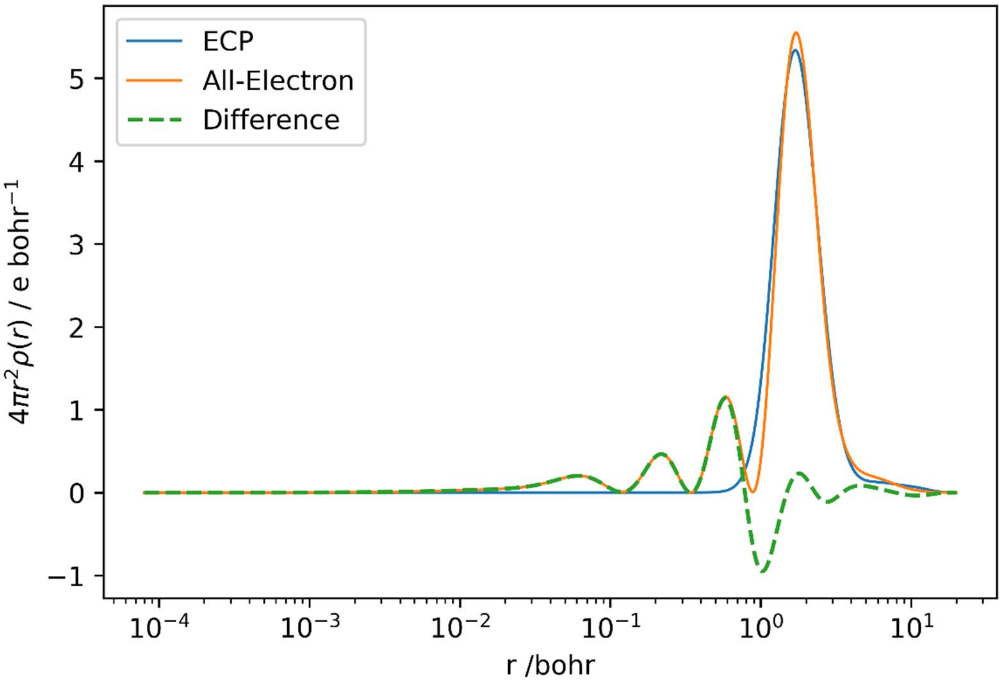

Figure S22 Difference in radial electron distribution function of 4s, 4p, 4d, 5s, 5p and 6s valence orbital for Cs using def2-TZVPP (Leininger *et al.*, 1996; Peterson *et al.*, 2003; Weigend & Ahlrichs, 2005; Gulde *et al.*, 2012; Dolg *et al.*, 1989; Andrae *et al.*, 1990) (blue), using an all-electron relativistic Jorge-TZVP-DKH calculation (orange) and the difference between the two distributions (green, dashed) against distance from the nucleus on a logarithmic scale.

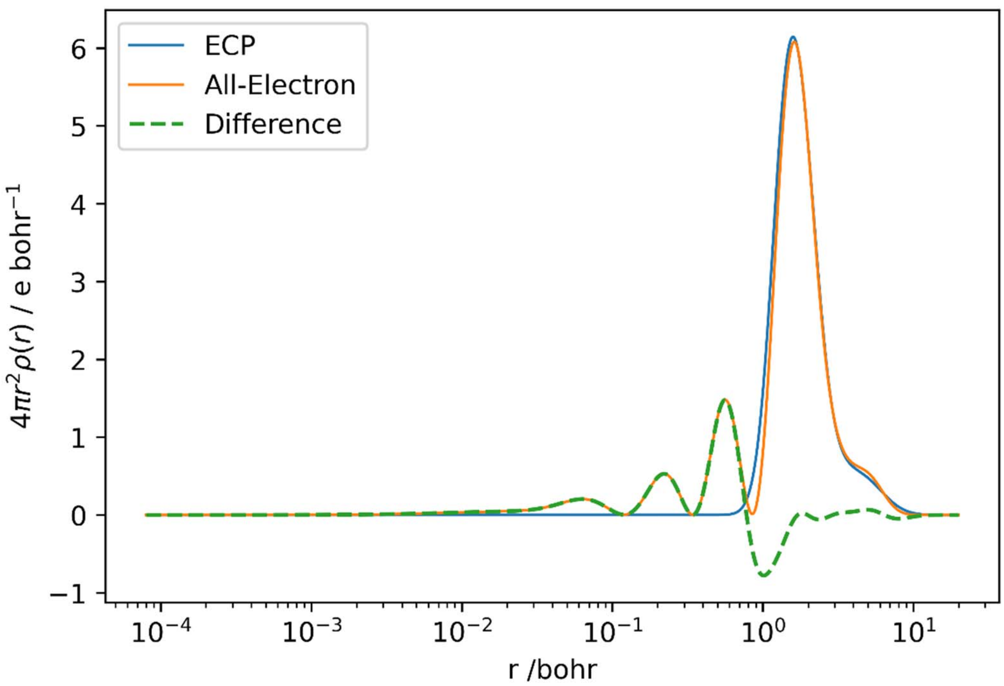

Figure S23 Difference in radial electron distribution function of 4s, 4p, 4d, 5s, 5p and 6s valence orbital for Ba using def2-TZVPP (Leininger *et al.*, 1996; Peterson *et al.*, 2003; Weigend & Ahlrichs, 2005; Gulde *et al.*, 2012; Dolg *et al.*, 1989; Andrae *et al.*, 1990) (blue), using an all-electron relativistic Jorge-TZVP-DKH calculation (orange) and the difference between the two distributions (green, dashed) against distance from the nucleus on a logarithmic scale.

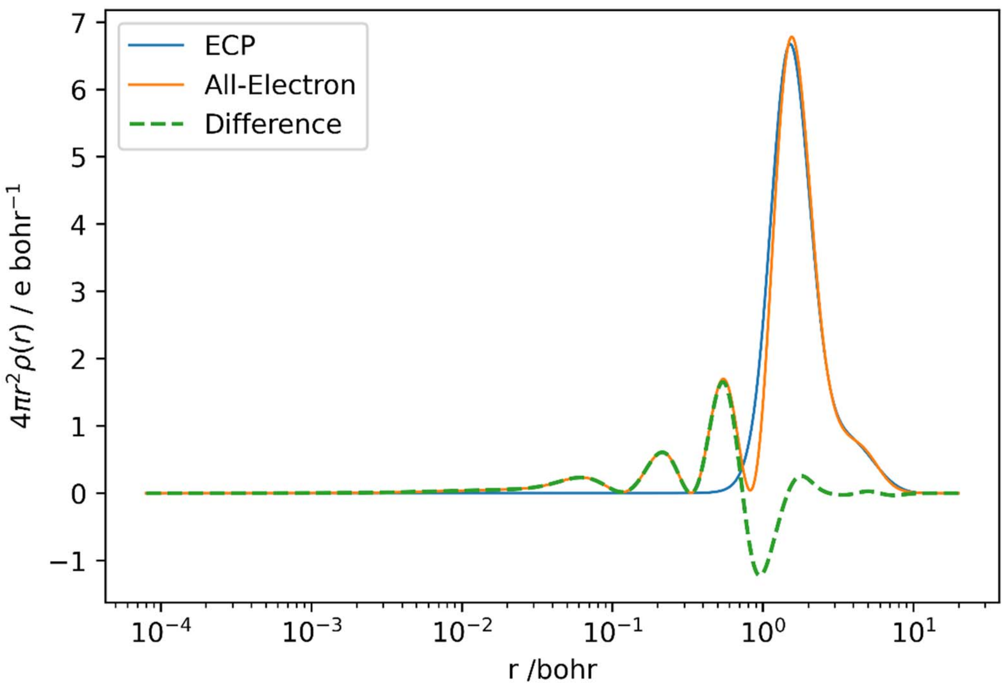

Figure S24 Difference in radial electron distribution function of 4s, 4p, 4d, 5s, 5p, 5d and 6s valence orbital for La using def2-TZVPP (Leininger *et al.*, 1996; Peterson *et al.*, 2003; Weigend & Ahlrichs, 2005; Gulde *et al.*, 2012; Dolg *et al.*, 1989; Andrae *et al.*, 1990) (blue), using an all-electron relativistic Jorge-TZVP-DKH calculation (orange) and the difference between the two distributions (green, dashed) against distance from the nucleus on a logarithmic scale.

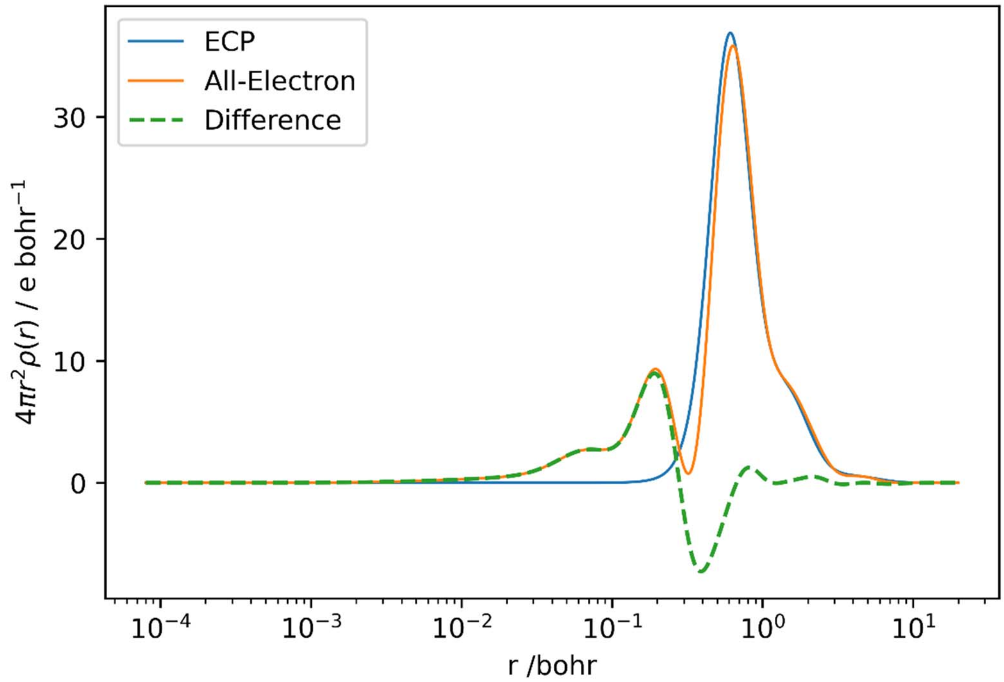

Figure S25 Difference in radial electron distribution function of 3s, 3p, 3d, 4s, 4p, 4d, 4f, 5s, 5p, 5d, and 6s valence orbital for Ce using def2-TZVPP (Leininger *et al.*, 1996; Peterson *et al.*, 2003; Weigend & Ahlrichs, 2005; Gulde *et al.*, 2012; Dolg *et al.*, 1989; Andrae *et al.*, 1990) (blue), using an all-electron relativistic Jorge-TZVP-DKH calculation (orange) and the difference between the two distributions (green, dashed) against distance from the nucleus on a logarithmic scale.

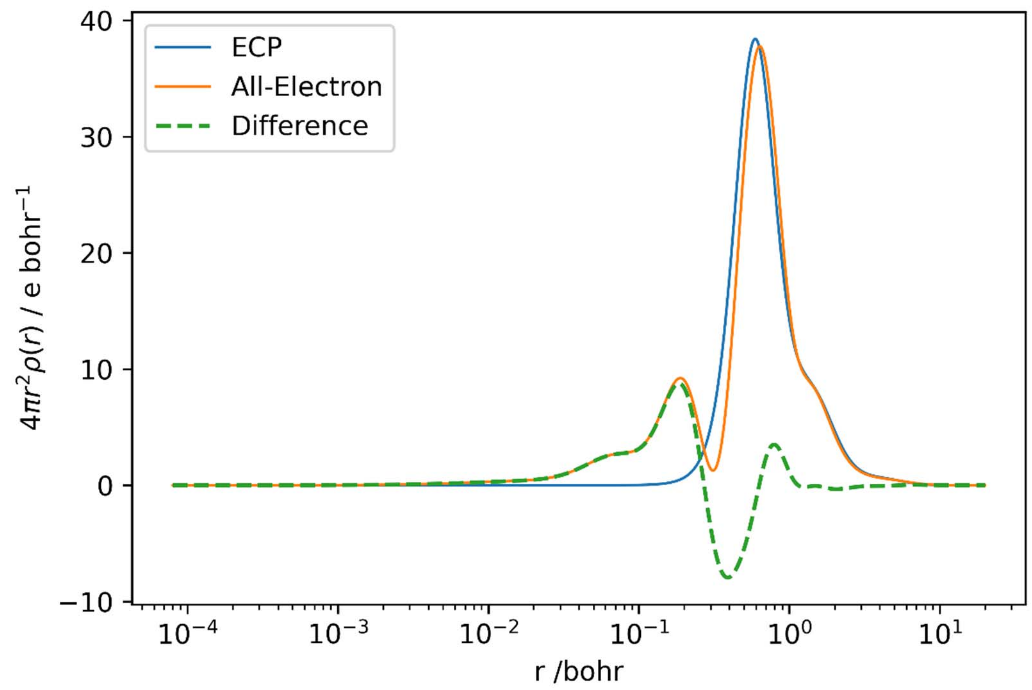

Figure S26 Difference in radial electron distribution function of 3s, 3p, 3d, 4s, 4p, 4d, 4f, 5s, 5p, 5d, and 6s valence orbital for Pr using def2-TZVPP (Leininger *et al.*, 1996; Peterson *et al.*, 2003; Weigend & Ahlrichs, 2005; Gulde *et al.*, 2012; Dolg *et al.*, 1989; Andrae *et al.*, 1990) (blue), using an all-electron relativistic Jorge-TZVP-DKH calculation (orange) and the difference between the two distributions (green, dashed) against distance from the nucleus on a logarithmic scale.

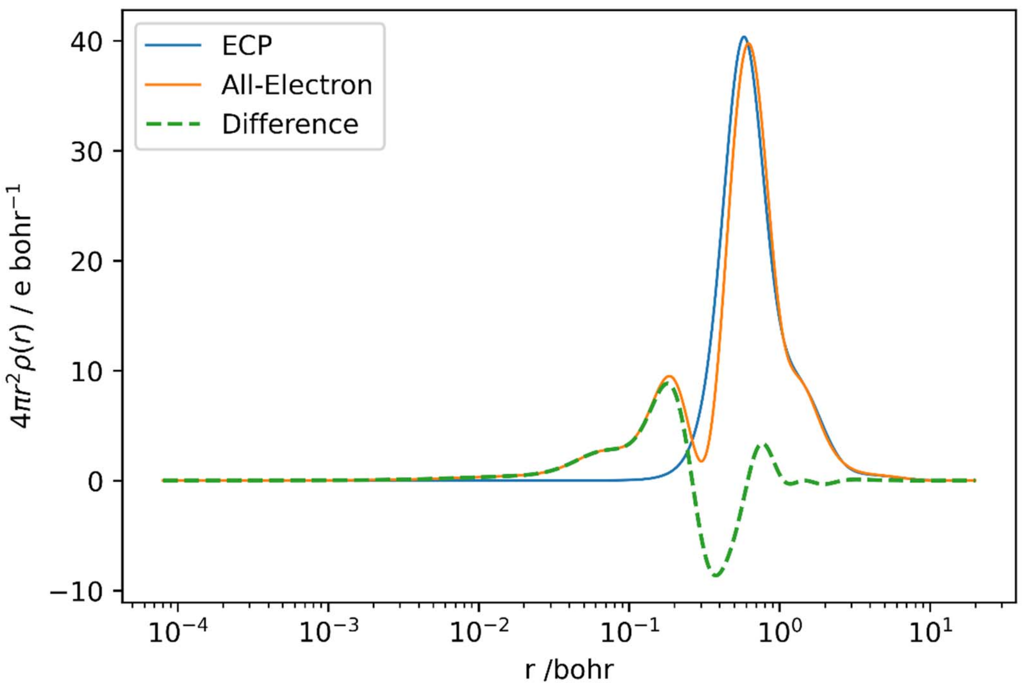

Figure S27 Difference in radial electron distribution function of 3s, 3p, 3d, 4s, 4p, 4d, 4f, 5s, 5p, 5d and 6s valence orbital for Nd using def2-TZVPP (Leininger *et al.*, 1996; Peterson *et al.*, 2003; Weigend & Ahlrichs, 2005; Gulde *et al.*, 2012; Dolg *et al.*, 1989; Andrae *et al.*, 1990) (blue), using an all-electron relativistic Jorge-TZVP-DKH calculation (orange) and the difference between the two distributions (green, dashed) against distance from the nucleus on a logarithmic scale.

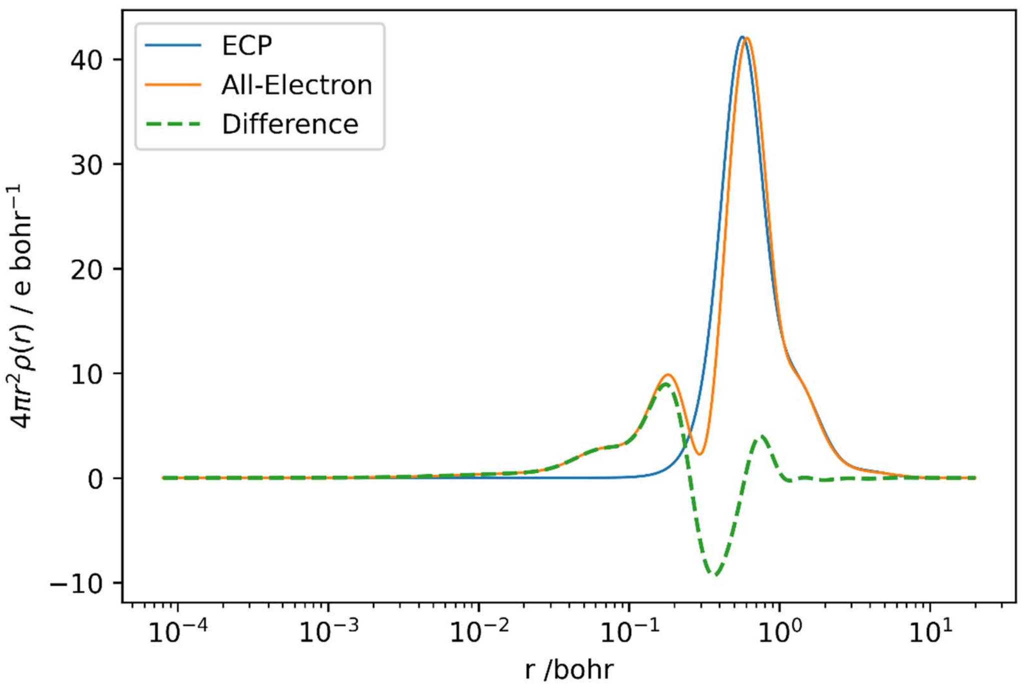

Figure S28 Difference in radial electron distribution function of 3s, 3p, 3d, 4s, 4p, 4d, 4f, 5s, 5p, 5d and 6s valence orbital for Pm using def2-TZVPP (Leininger *et al.*, 1996; Peterson *et al.*, 2003; Weigend & Ahlrichs, 2005; Gulde *et al.*, 2012; Dolg *et al.*, 1989; Andrae *et al.*, 1990) (blue), using an all-electron relativistic Jorge-TZVP-DKH calculation (orange) and the difference between the two distributions (green, dashed) against distance from the nucleus on a logarithmic scale.

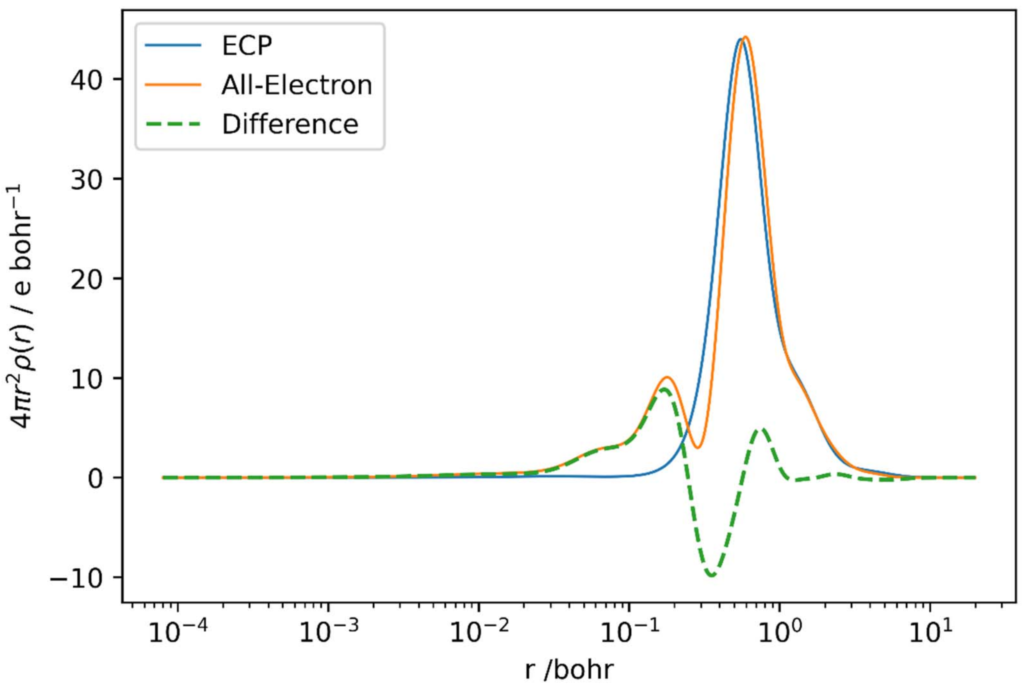

Figure S29 Difference in radial electron distribution function of 3s, 3p, 3d, 4s, 4p, 4d, 4f, 5s, 5p, 5d and 6s valence orbital for Sm using def2-TZVPP (Leininger *et al.*, 1996; Peterson *et al.*, 2003; Weigend & Ahlrichs, 2005; Gulde *et al.*, 2012; Dolg *et al.*, 1989; Andrae *et al.*, 1990) (blue), using an all-electron relativistic Jorge-TZVP-DKH calculation (orange) and the difference between the two distributions (green, dashed) against distance from the nucleus on a logarithmic scale.

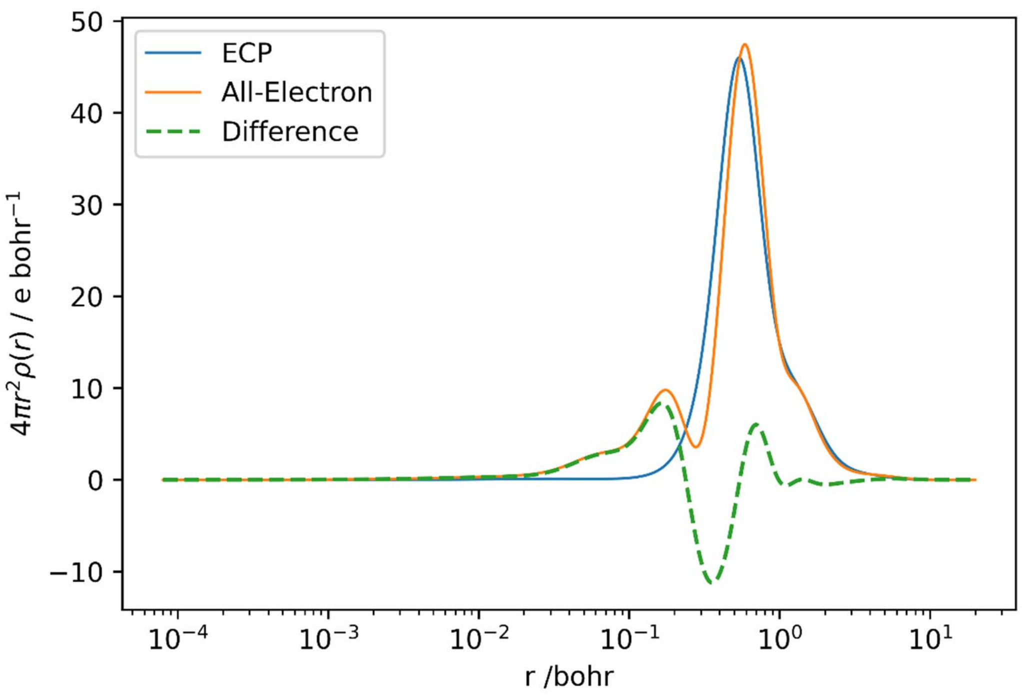

Figure S30 Difference in radial electron distribution function of 3s, 3p, 3d, 4s, 4p, 4d, 4f, 5s, 5p, 5d and 6s valence orbital for Eu using def2-TZVPP (Leininger *et al.*, 1996; Peterson *et al.*, 2003; Weigend & Ahlrichs, 2005; Gulde *et al.*, 2012; Dolg *et al.*, 1989; Andrae *et al.*, 1990) (blue), using an all-electron relativistic Jorge-TZVP-DKH calculation (orange) and the difference between the two distributions (green, dashed) against distance from the nucleus on a logarithmic scale.

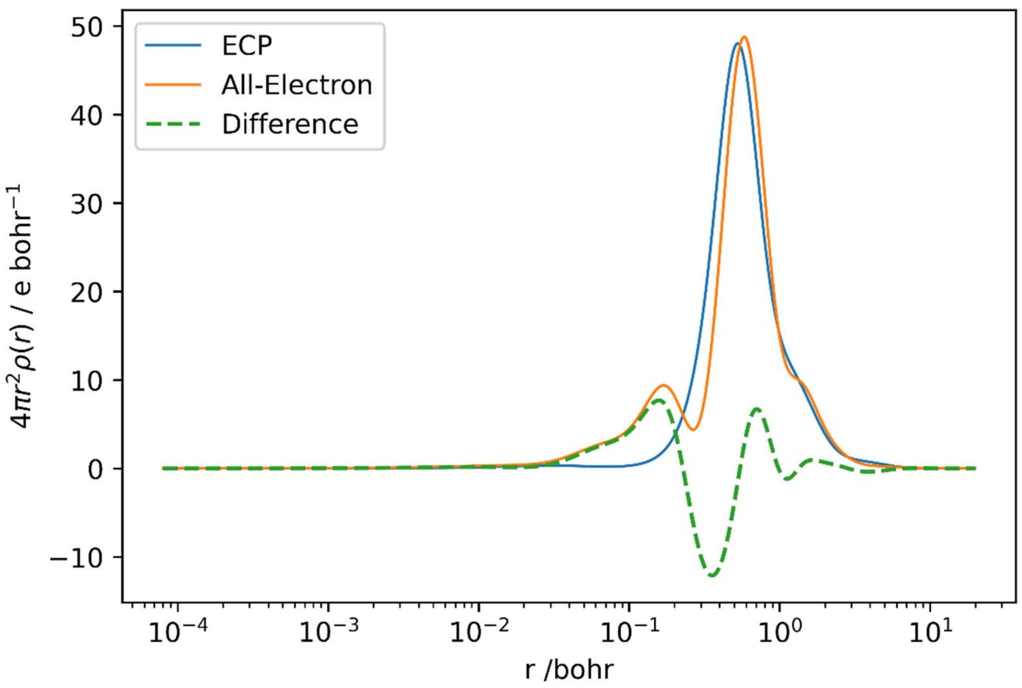

Figure S31 Difference in radial electron distribution function of 3s, 3p, 3d, 4s, 4p, 4d, 4f, 5s, 5p, 5d and 6s valence orbital for Gd using def2-TZVPP (Leininger *et al.*, 1996; Peterson *et al.*, 2003; Weigend & Ahlrichs, 2005; Gulde *et al.*, 2012; Dolg *et al.*, 1989; Andrae *et al.*, 1990) (blue), using an all-electron relativistic Jorge-TZVP-DKH calculation (orange) and the difference between the two distributions (green, dashed) against distance from the nucleus on a logarithmic scale.

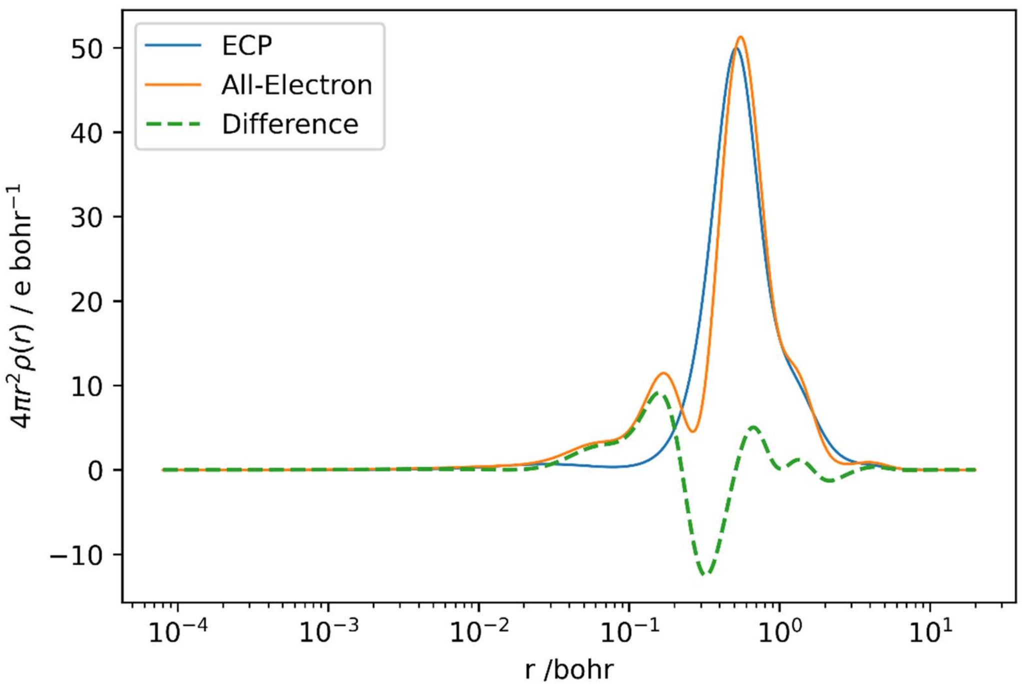

Figure S32 Difference in radial electron distribution function of 3s, 3p, 3d, 4s, 4p, 4d, 4f, 5s, 5p, 5d and 6s valence orbital for Tb using def2-TZVPP (Leininger *et al.*, 1996; Peterson *et al.*, 2003; Weigend & Ahlrichs, 2005; Gulde *et al.*, 2012; Dolg *et al.*, 1989; Andrae *et al.*, 1990) (blue), using an all-electron relativistic Jorge-TZVP-DKH calculation (orange) and the difference between the two distributions (green, dashed) against distance from the nucleus on a logarithmic scale.

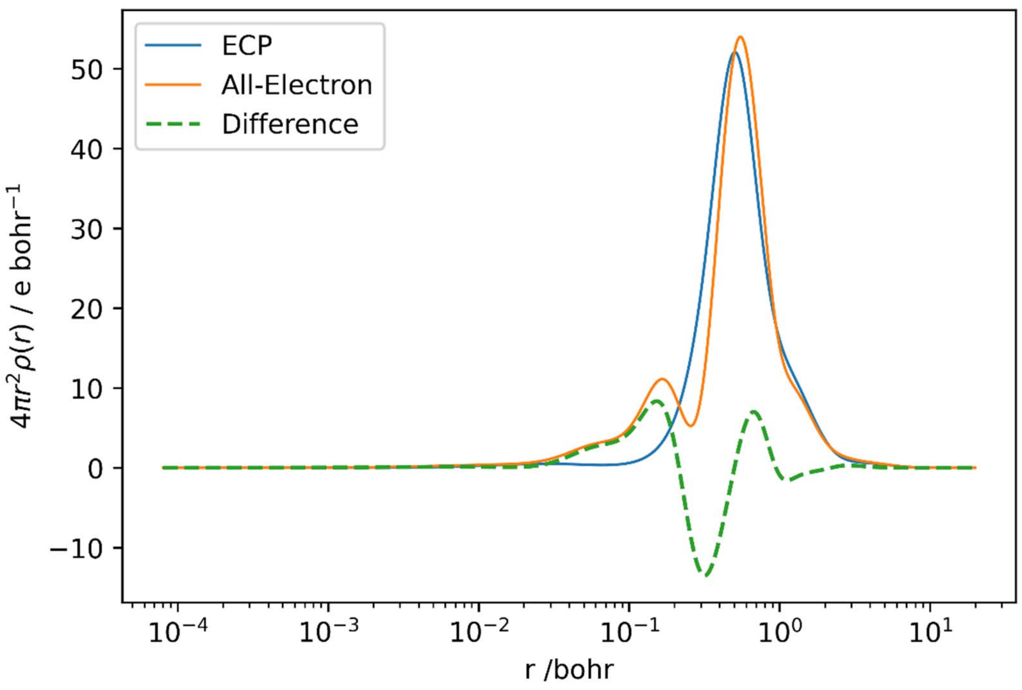

Figure S33 Difference in radial electron distribution function of 3s, 3p, 3d, 4s, 4p, 4d, 4f, 5s, 5p, 5d and 6s valence orbital for Dy using def2-TZVPP (Leininger *et al.*, 1996; Peterson *et al.*, 2003; Weigend & Ahlrichs, 2005; Gulde *et al.*, 2012; Dolg *et al.*, 1989; Andrae *et al.*, 1990) (blue), using an all-electron relativistic Jorge-TZVP-DKH calculation (orange) and the difference between the two distributions (green, dashed) against distance from the nucleus on a logarithmic scale.

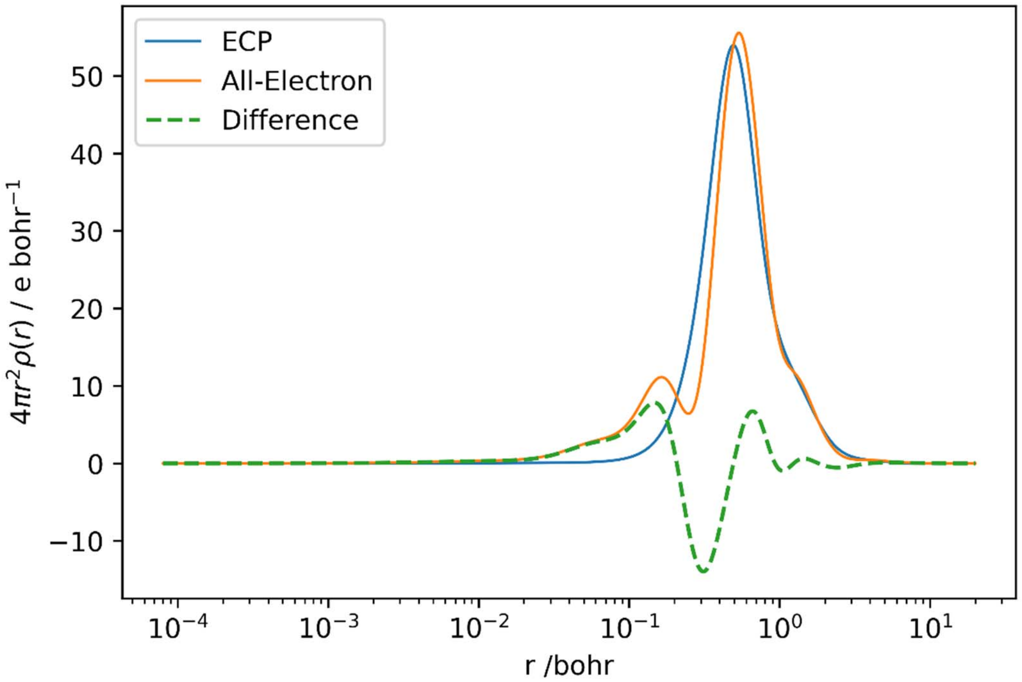

Figure S34 Difference in radial electron distribution function of 3s, 3p, 3d, 4s, 4p, 4d, 4f, 5s, 5p, 5d and 6s valence orbital for Ho using def2-TZVPP (Leininger *et al.*, 1996; Peterson *et al.*, 2003; Weigend & Ahlrichs, 2005; Gulde *et al.*, 2012; Dolg *et al.*, 1989; Andrae *et al.*, 1990) (blue), using an all-electron relativistic Jorge-TZVP-DKH calculation (orange) and the difference between the two distributions (green, dashed) against distance from the nucleus on a logarithmic scale.

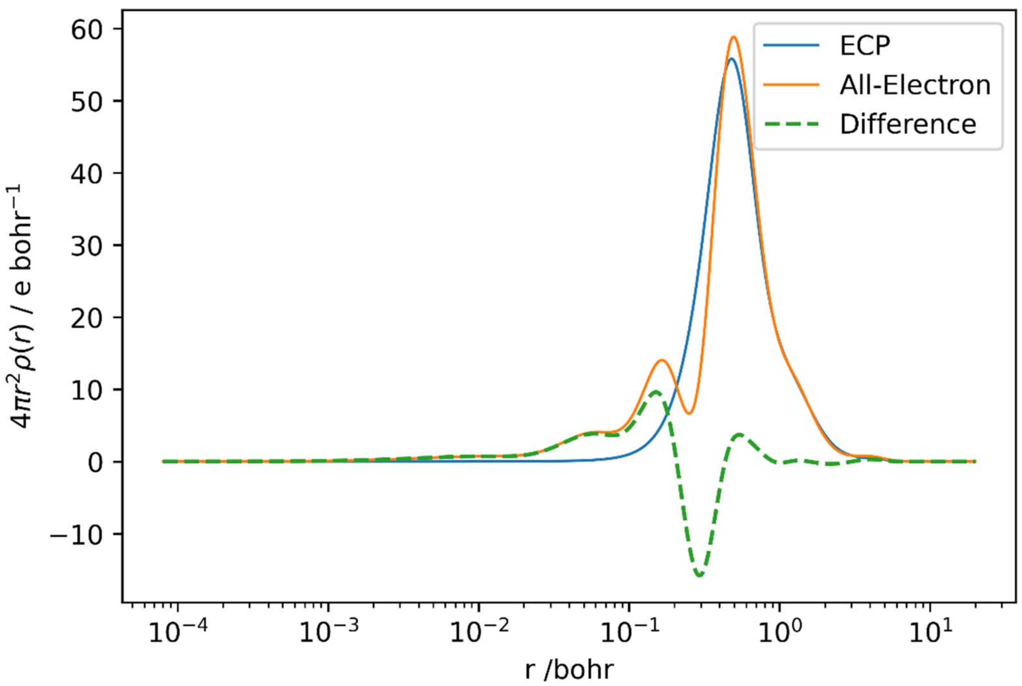

Figure S35 Difference in radial electron distribution function of 3s, 3p, 3d, 4s, 4p, 4d, 4f, 5s, 5p, 5d and 6s valence orbital for Er using def2-TZVPP (Leininger *et al.*, 1996; Peterson *et al.*, 2003; Weigend & Ahlrichs, 2005; Gulde *et al.*, 2012; Dolg *et al.*, 1989; Andrae *et al.*, 1990) (blue), using an all-electron relativistic Jorge-TZVP-DKH calculation (orange) and the difference between the two distributions (green, dashed) against distance from the nucleus on a logarithmic scale.

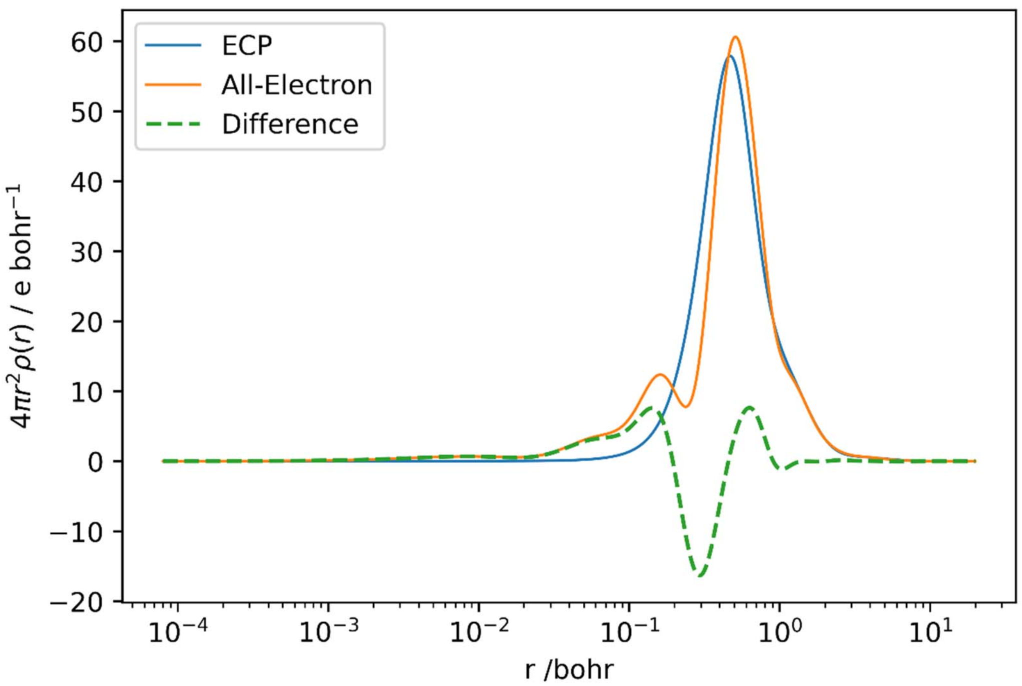

Figure S36 Difference in radial electron distribution function of 3s, 3p, 3d, 4s, 4p, 4d, 4f, 5s, 5p, 5d and 6s valence orbital for Tm using def2-TZVPP (Leininger *et al.*, 1996; Peterson *et al.*, 2003; Weigend & Ahlrichs, 2005; Gulde *et al.*, 2012; Dolg *et al.*, 1989; Andrae *et al.*, 1990) (blue), using an all-electron relativistic Jorge-TZVP-DKH calculation (orange) and the difference between the two distributions (green, dashed) against distance from the nucleus on a logarithmic scale.

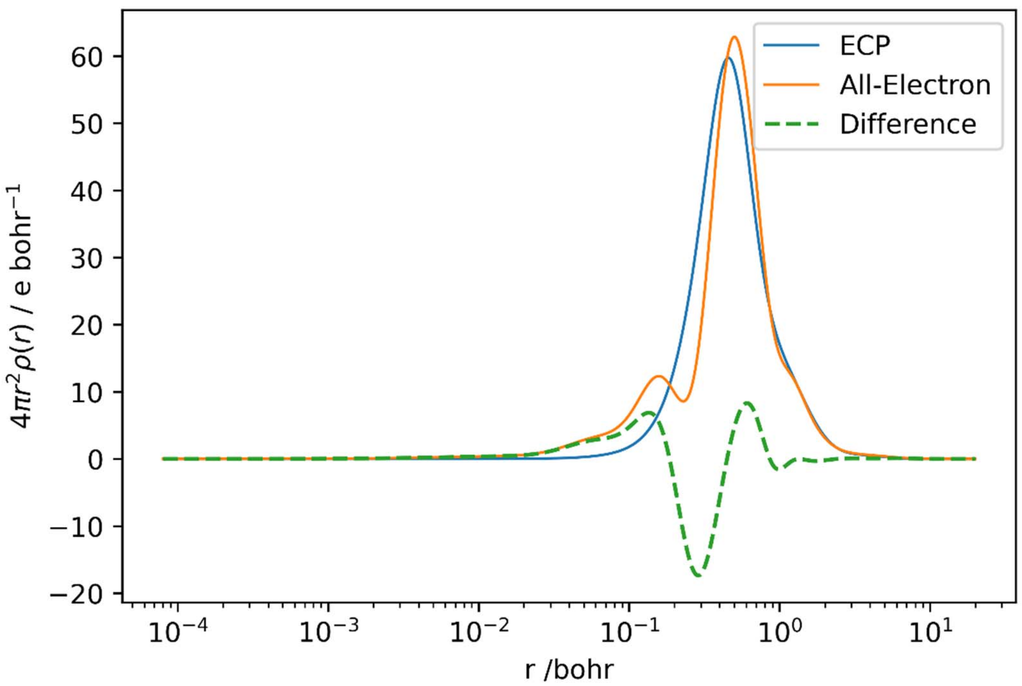

Figure S37 Difference in radial electron distribution function of 3s, 3p, 3d, 4s, 4p, 4d, 4f, 5s, 5p, 5d and 6s valence orbital for Yb using def2-TZVPP (Leininger *et al.*, 1996; Peterson *et al.*, 2003; Weigend & Ahlrichs, 2005; Gulde *et al.*, 2012; Dolg *et al.*, 1989; Andrae *et al.*, 1990) (blue), using an all-electron relativistic Jorge-TZVP-DKH calculation (orange) and the difference between the two distributions (green, dashed) against distance from the nucleus on a logarithmic scale.

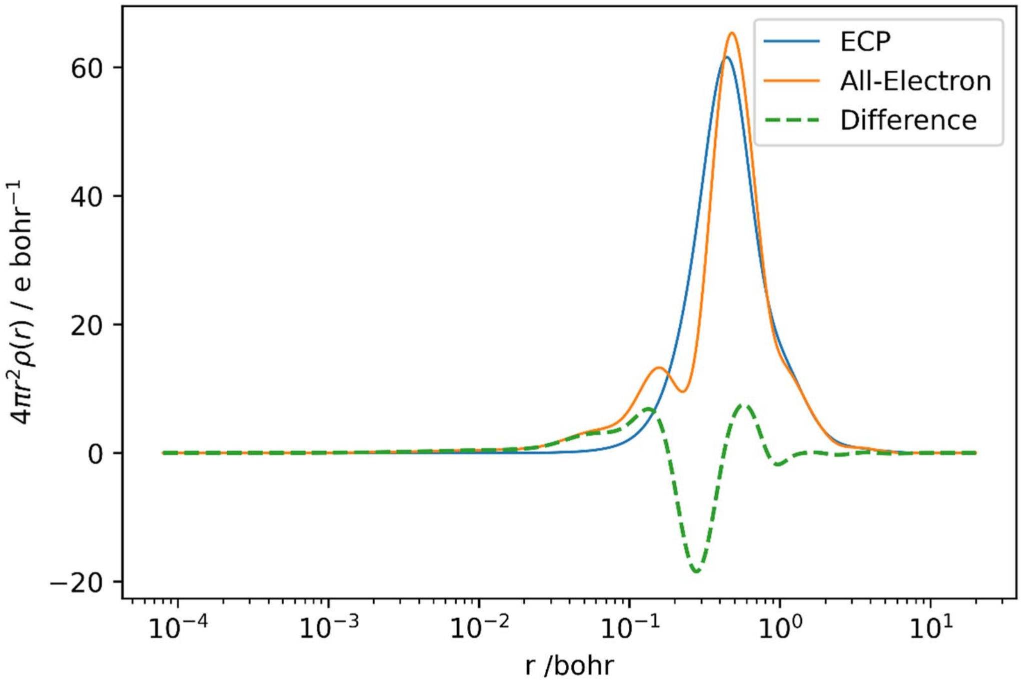

Figure S38 Difference in radial electron distribution function of 3s, 3p, 3d, 4s, 4p, 4d, 4f, 5s, 5p, 5d, and 6s valence orbital for Lu using def2-TZVPP (Leininger *et al.*, 1996; Peterson *et al.*, 2003; Weigend & Ahlrichs, 2005; Gulde *et al.*, 2012; Dolg *et al.*, 1989; Andrae *et al.*, 1990) (blue), using an all-electron relativistic Jorge-TZVP-DKH calculation (orange) and the difference between the two distributions (green, dashed) against distance from the nucleus on a logarithmic scale.

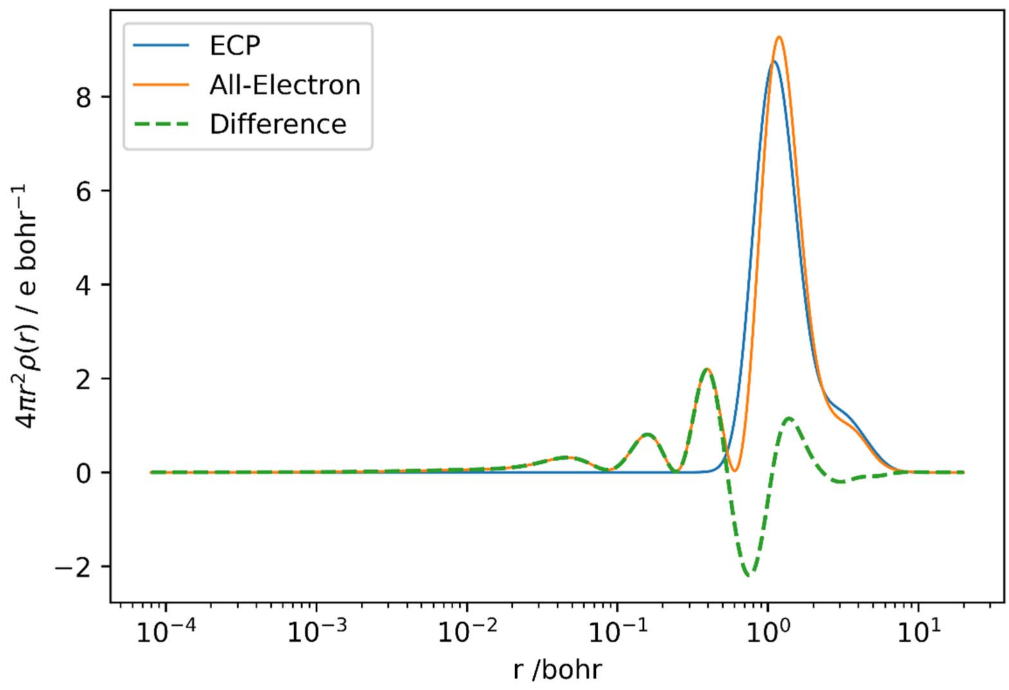

Figure S39 Difference in radial electron distribution function of 5s, 5p, 5d, and 6s valence orbital for Hf using def2-TZVPP (Leininger *et al.*, 1996; Peterson *et al.*, 2003; Weigend & Ahlrichs, 2005; Gulde *et al.*, 2012; Dolg *et al.*, 1989; Andrae *et al.*, 1990) (blue), using an all-electron relativistic Jorge-TZVP-DKH calculation (orange) and the difference between the two distributions (green, dashed) against distance from the nucleus on a logarithmic scale.

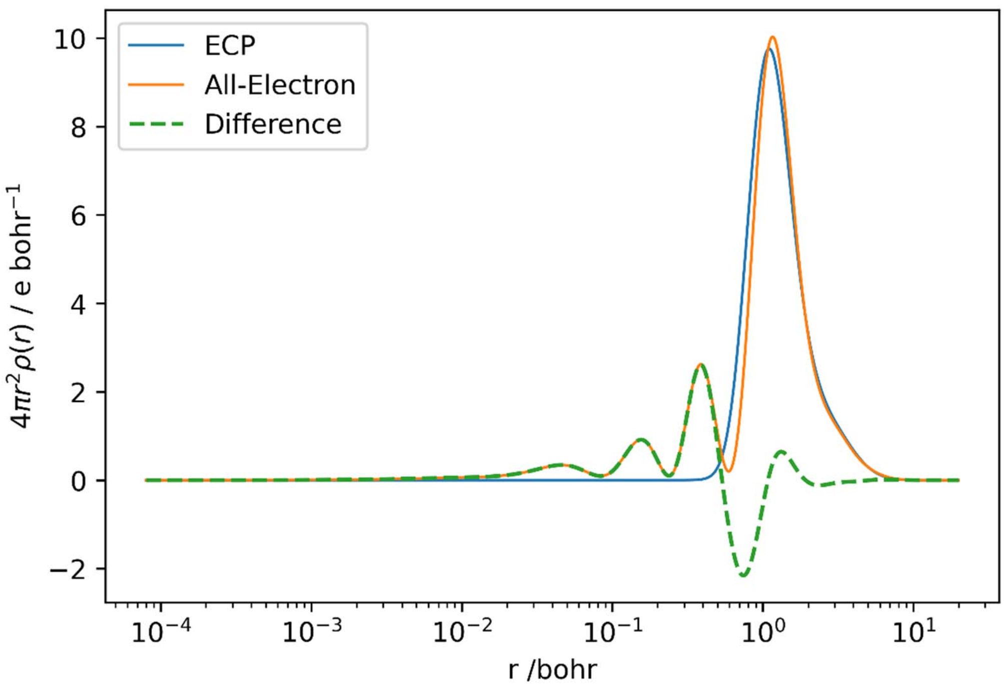

Figure S40 Difference in radial electron distribution function of 5s, 5p, 5d, and 6s valence orbital for Ta using def2-TZVPP (Leininger *et al.*, 1996; Peterson *et al.*, 2003; Weigend & Ahlrichs, 2005; Gulde *et al.*, 2012; Dolg *et al.*, 1989; Andrae *et al.*, 1990) (blue), using an all-electron relativistic Jorge-TZVP-DKH calculation (orange) and the difference between the two distributions (green, dashed) against distance from the nucleus on a logarithmic scale.

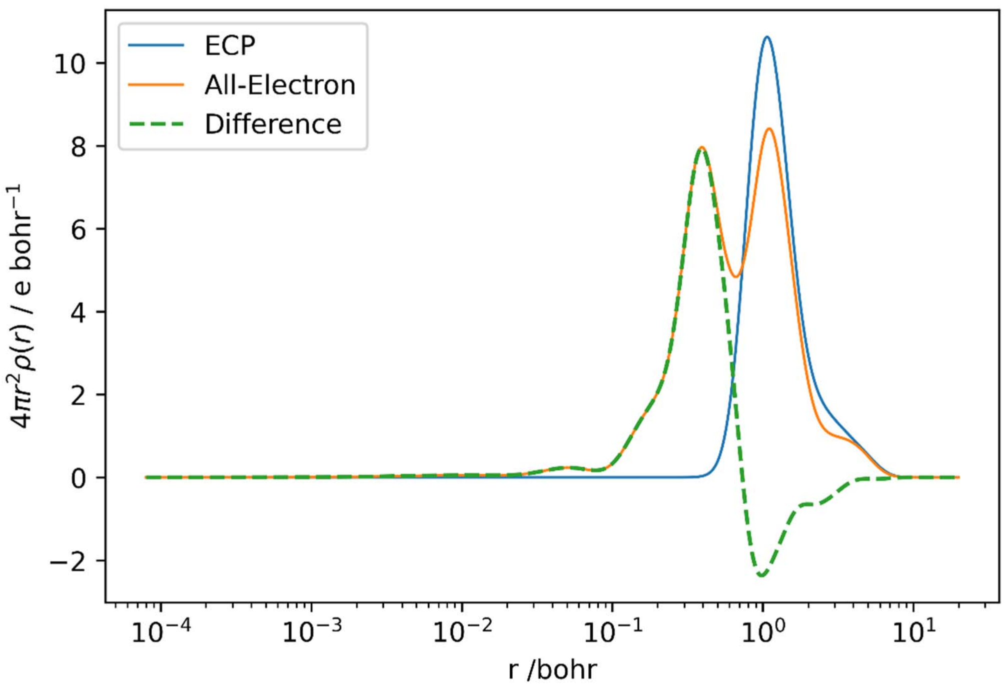

Figure S41 Difference in radial electron distribution function of 5s, 5p, 5d, and 6s valence orbital for W using def2-TZVPP (Leininger *et al.*, 1996; Peterson *et al.*, 2003; Weigend & Ahlrichs, 2005; Gulde *et al.*, 2012; Dolg *et al.*, 1989; Andrae *et al.*, 1990) (blue), using an all-electron relativistic Jorge-TZVP-DKH calculation (orange) and the difference between the two distributions (green, dashed) against distance from the nucleus on a logarithmic scale.

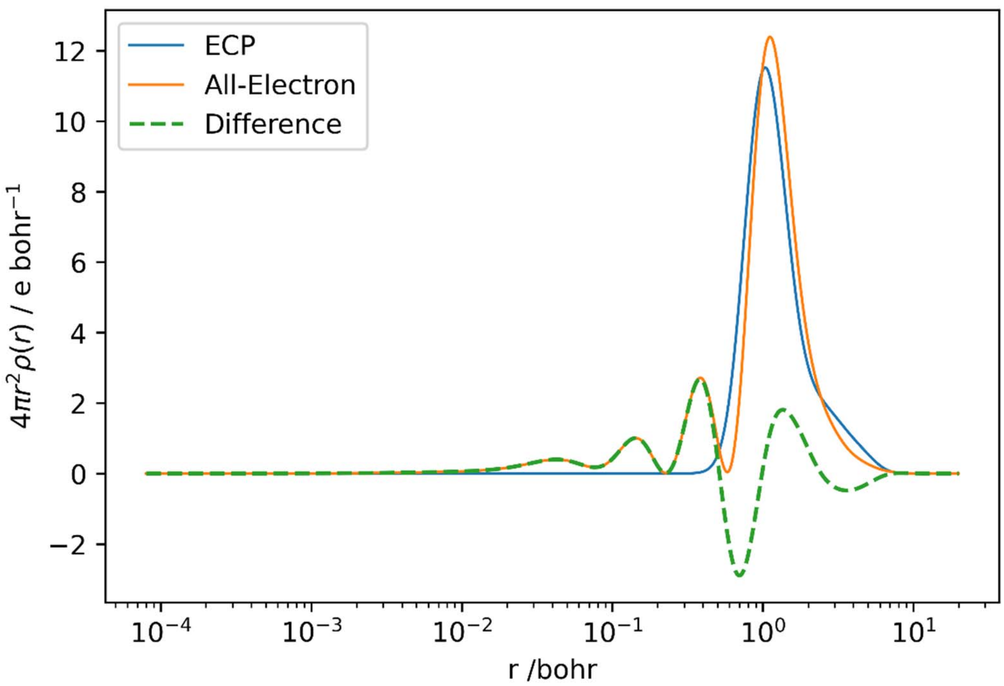

Figure S42 Difference in radial electron distribution function of 5s, 5p, 5d, and 6s valence orbital for Re using def2-TZVPP (Leininger *et al.*, 1996; Peterson *et al.*, 2003; Weigend & Ahlrichs, 2005; Gulde *et al.*, 2012; Dolg *et al.*, 1989; Andrae *et al.*, 1990) (blue), using an all-electron relativistic Jorge-TZVP-DKH calculation (orange) and the difference between the two distributions (green, dashed) against distance from the nucleus on a logarithmic scale.

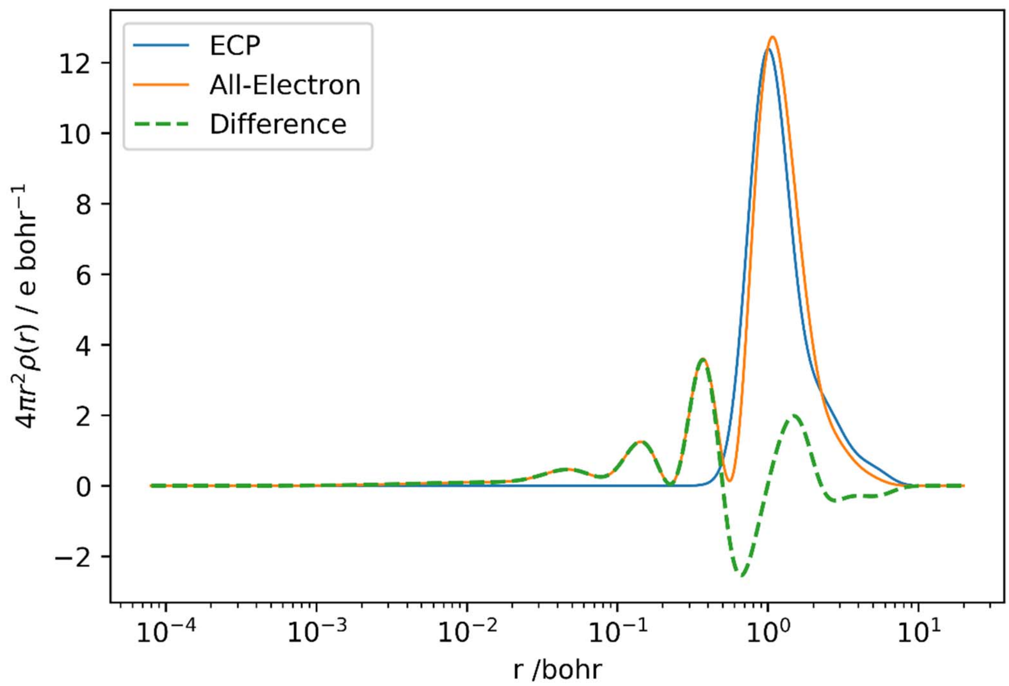

Figure S43 Difference in radial electron distribution function of 5s, 5p, 5d, and 6s valence orbital for Os using def2-TZVPP (Leininger *et al.*, 1996; Peterson *et al.*, 2003; Weigend & Ahlrichs, 2005; Gulde *et al.*, 2012; Dolg *et al.*, 1989; Andrae *et al.*, 1990) (blue), using an all-electron relativistic Jorge-TZVP-DKH calculation (orange) and the difference between the two distributions (green, dashed) against distance from the nucleus on a logarithmic scale.

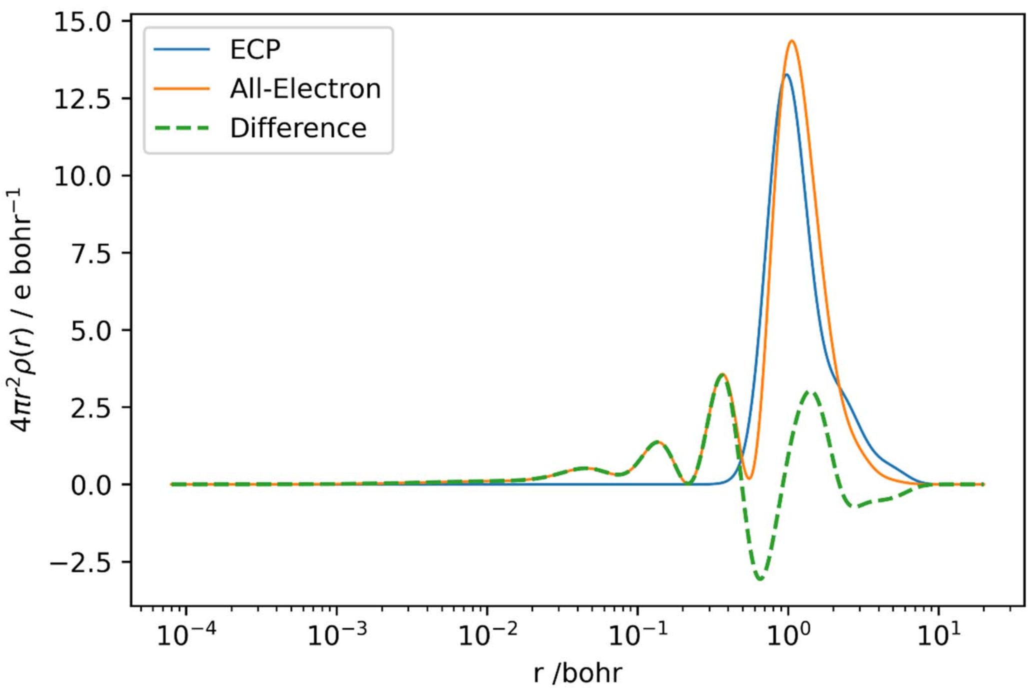

Figure S44 Difference in radial electron distribution function of 5s, 5p, 5d, and 6s valence orbital for Ir using def2-TZVPP (Leininger *et al.*, 1996; Peterson *et al.*, 2003; Weigend & Ahlrichs, 2005; Gulde *et al.*, 2012; Dolg *et al.*, 1989; Andrae *et al.*, 1990) (blue), using an all-electron relativistic Jorge-TZVP-DKH calculation (orange) and the difference between the two distributions (green, dashed) against distance from the nucleus on a logarithmic scale.

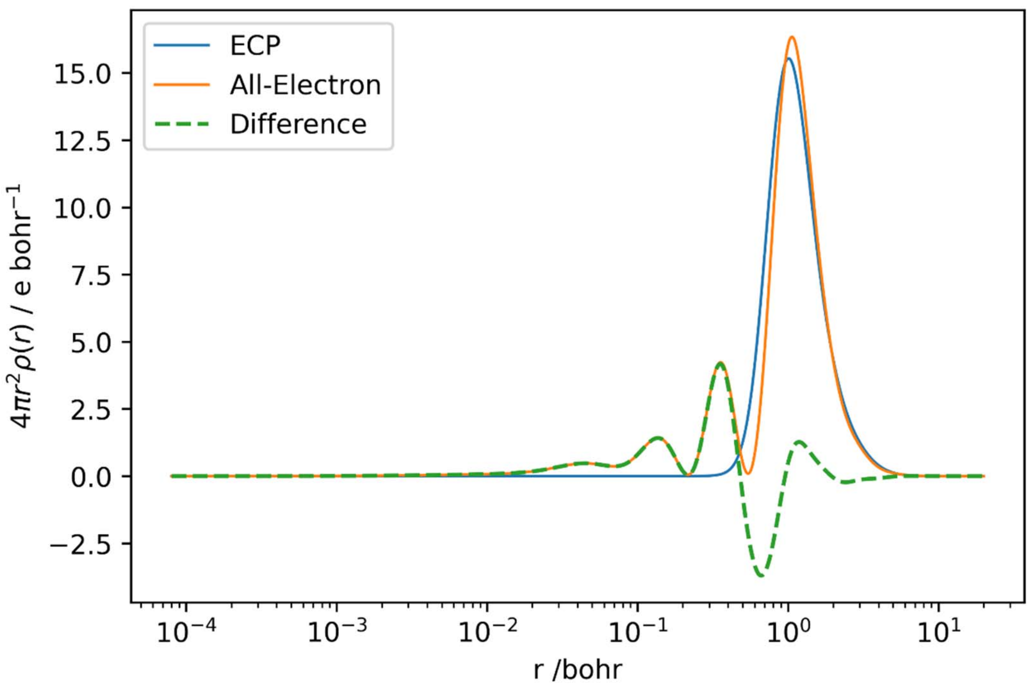

Figure S45 Difference in radial electron distribution function of 5s, 5p, 5d, and 6s valence orbital for Pt using def2-TZVPP (Leininger *et al.*, 1996; Peterson *et al.*, 2003; Weigend & Ahlrichs, 2005; Gulde *et al.*, 2012; Dolg *et al.*, 1989; Andrae *et al.*, 1990) (blue), using an all-electron relativistic Jorge-TZVP-DKH calculation (orange) and the difference between the two distributions (green, dashed) against distance from the nucleus on a logarithmic scale.

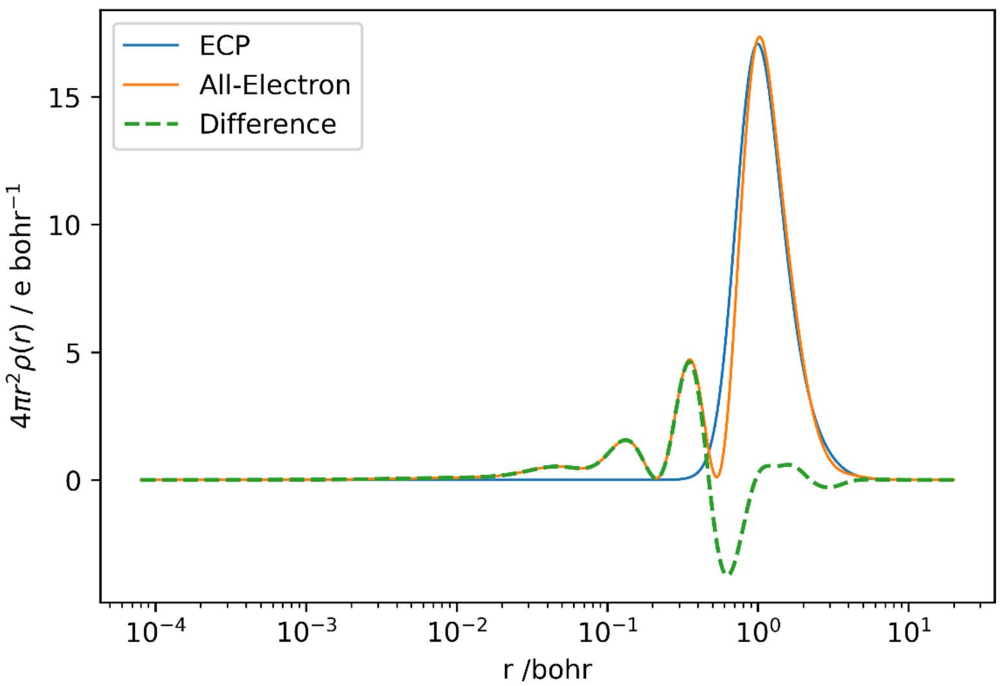

Figure S46 Difference in radial electron distribution function of 5s, 5p, 5d, and 6s valence orbital for Au using def2-TZVPP (Leininger *et al.*, 1996; Peterson *et al.*, 2003; Weigend & Ahlrichs, 2005; Gulde *et al.*, 2012; Dolg *et al.*, 1989; Andrae *et al.*, 1990) (blue), using an all-electron relativistic Jorge-TZVP-DKH calculation (orange) and the difference between the two distributions (green, dashed) against distance from the nucleus on a logarithmic scale.

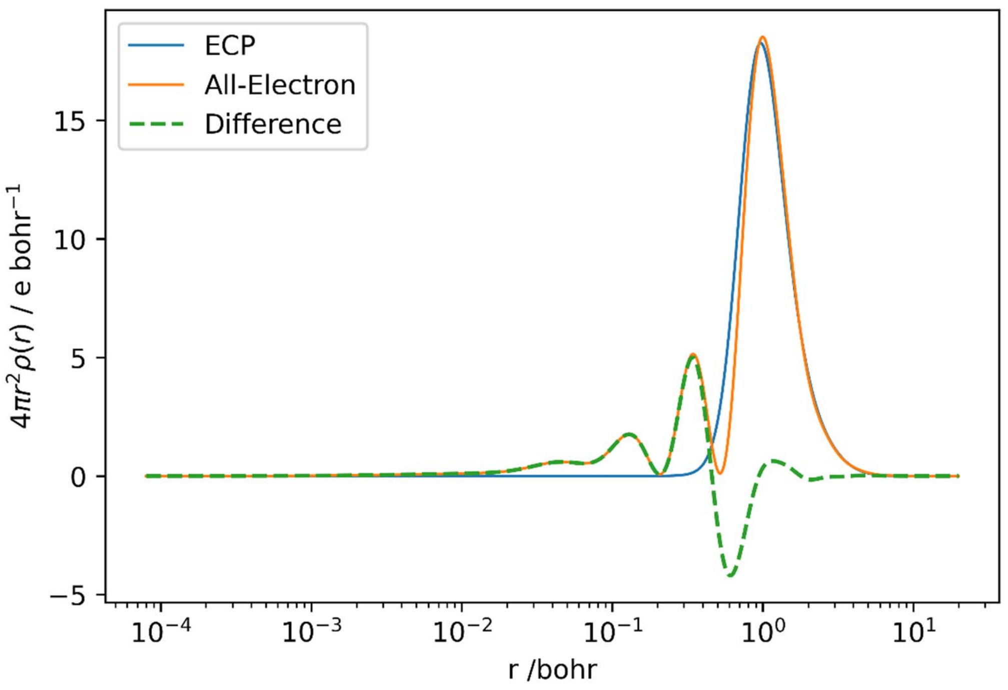

Figure S47 Difference in radial electron distribution function of 5s, 5p, 5d, and 6s valence orbital for Hg using def2-TZVPP (Leininger *et al.*, 1996; Peterson *et al.*, 2003; Weigend & Ahlrichs, 2005; Gulde *et al.*, 2012; Dolg *et al.*, 1989; Andrae *et al.*, 1990) (blue), using an all-electron relativistic Jorge-TZVP-DKH calculation (orange) and the difference between the two distributions (green, dashed) against distance from the nucleus on a logarithmic scale.

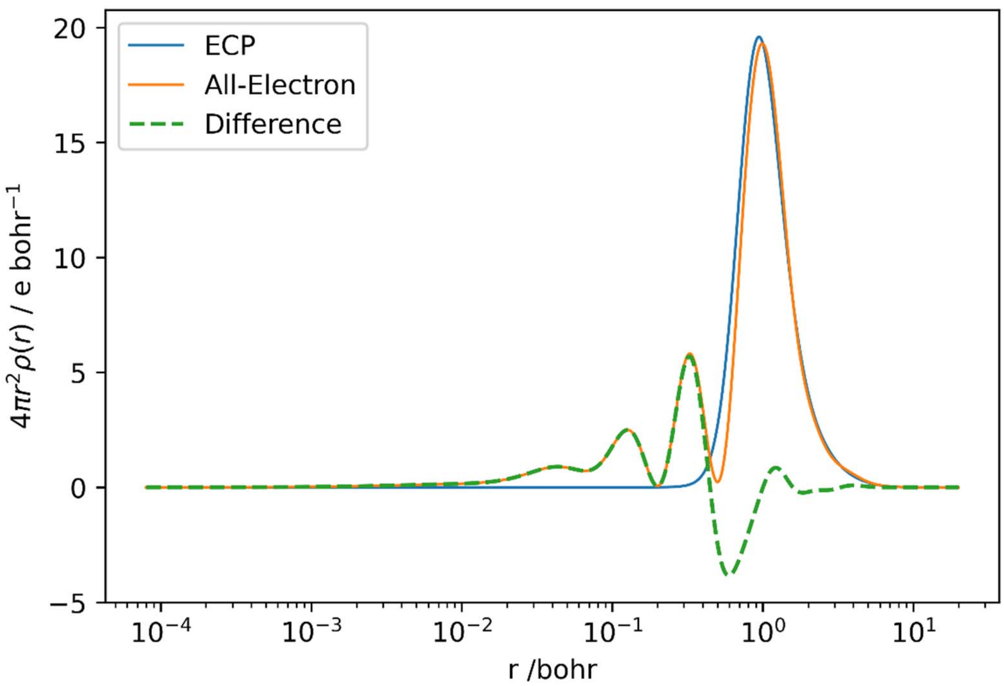

Figure S48 Difference in radial electron distribution function of 5s, 5p, 5d, 6s, and 6p valence orbital for Tl using def2-TZVPP (Leininger *et al.*, 1996; Peterson *et al.*, 2003; Weigend & Ahlrichs, 2005; Gulde *et al.*, 2012; Dolg *et al.*, 1989; Andrae *et al.*, 1990) (blue), using an all-electron relativistic Jorge-TZVP-DKH calculation (orange) and the difference between the two distributions (green, dashed) against distance from the nucleus on a logarithmic scale.

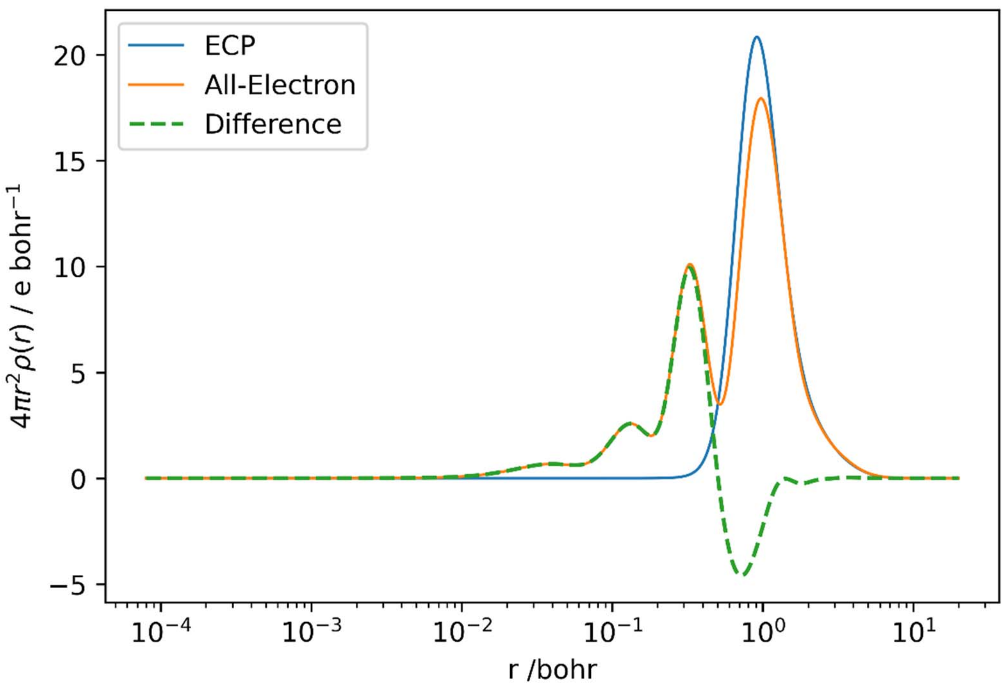

Figure S49 Difference in radial electron distribution function of 5s, 5p, 5d, 6s, and 6p valence orbital for Pb using def2-TZVPP (Leininger *et al.*, 1996; Peterson *et al.*, 2003; Weigend & Ahlrichs, 2005; Gulde *et al.*, 2012; Dolg *et al.*, 1989; Andrae *et al.*, 1990) (blue), using an all-electron relativistic Jorge-TZVP-DKH calculation (orange) and the difference between the two distributions (green, dashed) against distance from the nucleus on a logarithmic scale.

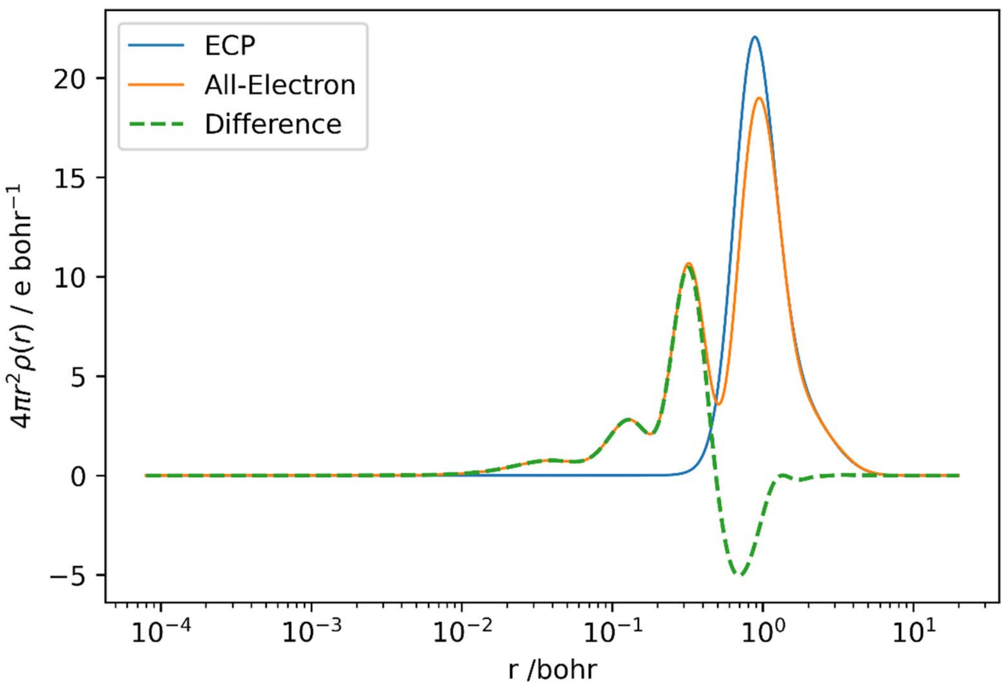

Figure S50 Difference in radial electron distribution function of 5s, 5p, 5d, 6s, and 6p valence orbital for Bi using def2-TZVPP (Leininger *et al.*, 1996; Peterson *et al.*, 2003; Weigend & Ahlrichs, 2005; Gulde *et al.*, 2012; Dolg *et al.*, 1989; Andrae *et al.*, 1990) (blue), using an all-electron relativistic Jorge-TZVP-DKH calculation (orange) and the difference between the two distributions (green, dashed) against distance from the nucleus on a logarithmic scale.

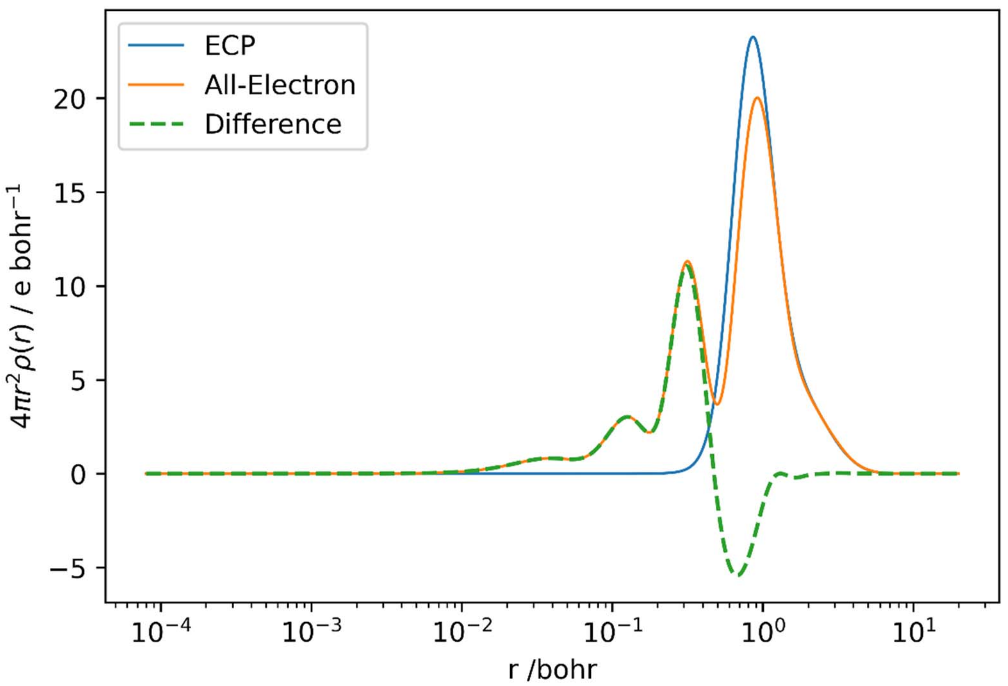

Figure S51 Difference in radial electron distribution function of 5s, 5p, 5d, 6s, and 6p valence orbital for Po using def2-TZVPP (Leininger *et al.*, 1996; Peterson *et al.*, 2003; Weigend & Ahlrichs, 2005; Gulde *et al.*, 2012; Dolg *et al.*, 1989; Andrae *et al.*, 1990) (blue), using an all-electron relativistic Jorge-TZVP-DKH calculation (orange) and the difference between the two distributions (green, dashed) against distance from the nucleus on a logarithmic scale.

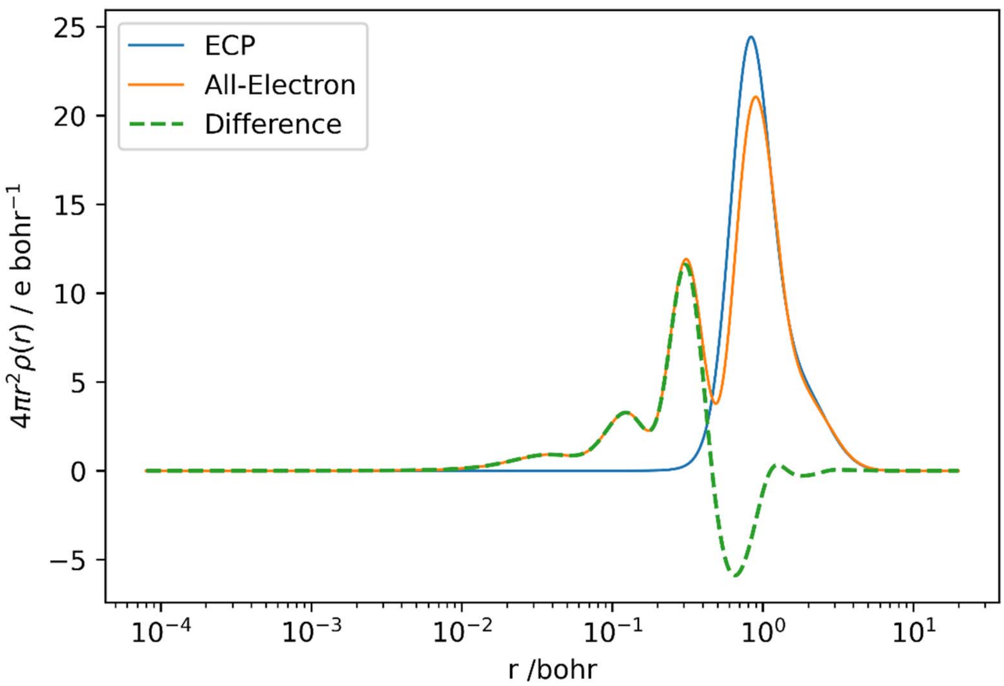

Figure S52 Difference in radial electron distribution function of 5s, 5p, 5d, 6s, and 6p valence orbital for At using def2-TZVPP (Leininger *et al.*, 1996; Peterson *et al.*, 2003; Weigend & Ahlrichs, 2005; Gulde *et al.*, 2012; Dolg *et al.*, 1989; Andrae *et al.*, 1990) (blue), using an all-electron relativistic Jorge-TZVP-DKH calculation (orange) and the difference between the two distributions (green, dashed) against distance from the nucleus on a logarithmic scale.

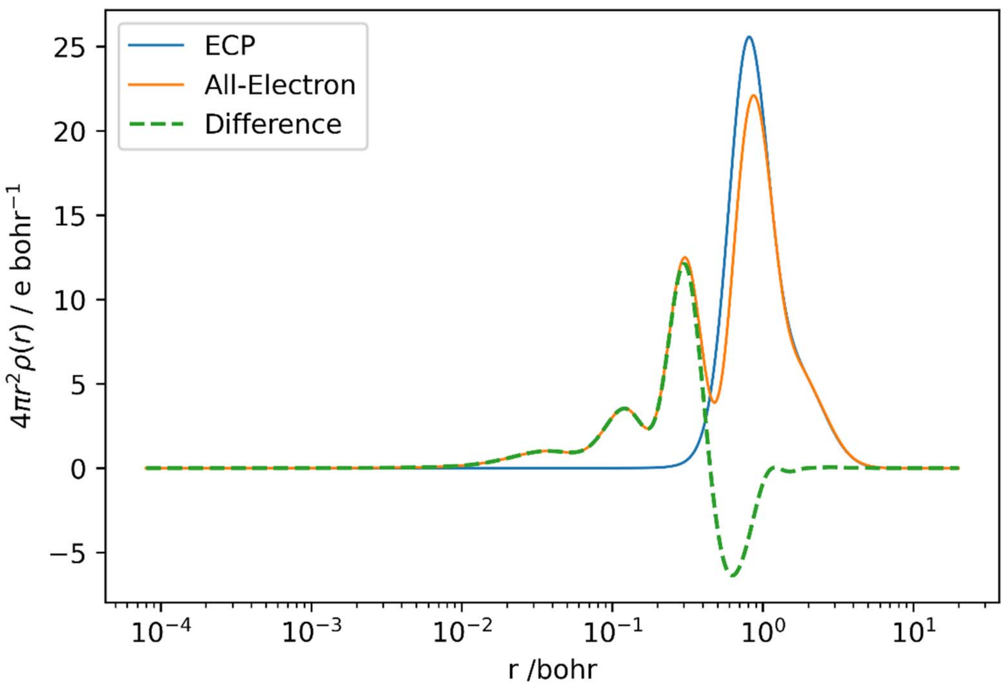

Figure S53 Difference in radial electron distribution function of 5s, 5p, 5d, 6s, and 6s valence orbital for Rn using def2-TZVPP (Leininger *et al.*, 1996; Peterson *et al.*, 2003; Weigend & Ahlrichs, 2005; Gulde *et al.*, 2012; Dolg *et al.*, 1989; Andrae *et al.*, 1990) (blue), using an all-electron relativistic Jorge-TZVP-DKH calculation (orange) and the difference between the two distributions (green, dashed) against distance from the nucleus on a logarithmic scale.

S5. Deformation Density Plots of the AuI_4^- Deformation Density

To investigate effects between the different models (all electron and ECP based) we include here a 2D-map of the deformation density within the Au-I plane of $[(Ph_3PO)_2H][AuI_4]$.

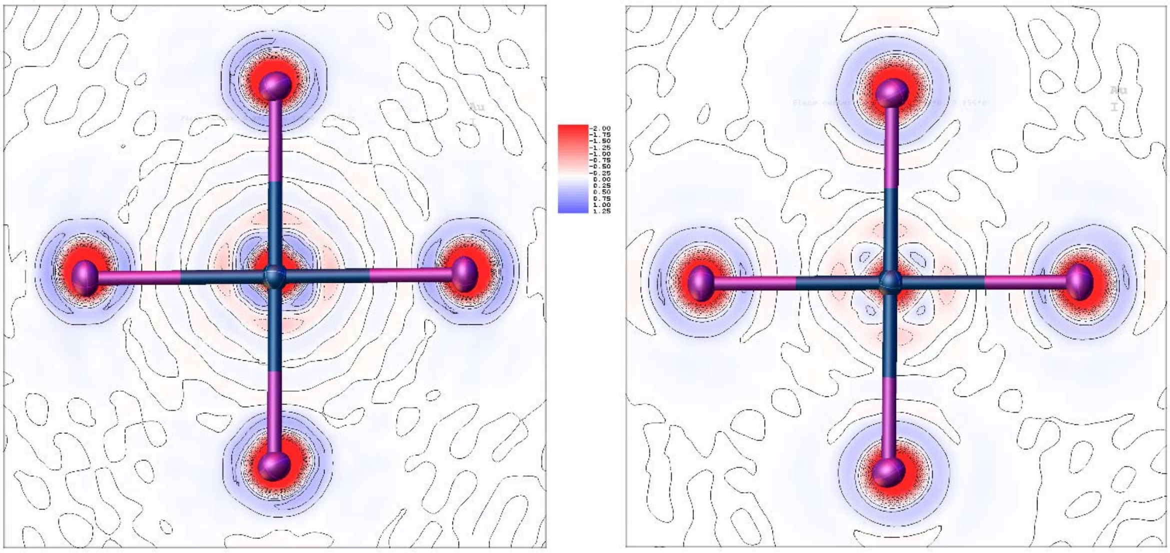

Figure S54 Deformation density maps of the AuI_4^- Anion within the molecular plane of the ECP based calculation (left) and the all electron calculation (right). Colour code is included between the maps and identical between both maps.
